# Combined small-molecule treatment accelerates maturation of human pluripotent stem cell-derived neurons

**DOI:** 10.1038/s41587-023-02031-z

**Published:** 2024-01-02

**Authors:** Emiliano Hergenreder, Andrew P. Minotti, Yana Zorina, Polina Oberst, Zeping Zhao, Hermany Munguba, Elizabeth L. Calder, Arianna Baggiolini, Ryan M. Walsh, Conor Liston, Joshua Levitz, Ralph Garippa, Shuibing Chen, Gabriele Ciceri, Lorenz Studer

**Affiliations:** 1 The Center for Stem Cell Biology, Sloan–Kettering Institute for Cancer Research, New York, NY USA; 2 Developmental Biology Program, Sloan–Kettering Institute for Cancer Research, New York, NY USA; 3 Weill Graduate School of Medical Sciences of Cornell University, New York, NY USA; 4 Gene Editing and Screening Core Facility, Sloan–Kettering Institute for Cancer Research, New York, NY USA; 5 Department of Surgery, Weill Cornell Medicine, New York, NY USA; 6 Department of Biochemistry, Weill Cornell Medicine, New York, NY USA; 7 Department of Psychiatry, Weill Cornell Medicine, New York, USA

**Keywords:** Embryonic stem cells, Induced pluripotent stem cells, Differentiation, Cellular neuroscience, Neuronal development

## Abstract

The maturation of human pluripotent stem cell (hPSC)-derived neurons mimics the protracted timing of human brain development, extending over months to years for reaching adult-like function. Prolonged in vitro maturation presents a major challenge to stem cell-based applications in modeling and treating neurological disease. Therefore, we designed a high-content imaging assay based on morphological and functional readouts in hPSC-derived cortical neurons which identified multiple compounds that drive neuronal maturation including inhibitors of lysine-specific demethylase 1 and disruptor of telomerase-like 1 and activators of calcium-dependent transcription. A cocktail of four factors, GSK2879552, EPZ-5676, *N*-methyl-d-aspartate and Bay K 8644, collectively termed GENtoniK, triggered maturation across all parameters tested, including synaptic density, electrophysiology and transcriptomics. Maturation effects were further validated in cortical organoids, spinal motoneurons and non-neural lineages including melanocytes and pancreatic β-cells. The effects on maturation observed across a broad range of hPSC-derived cell types indicate that some of the mechanisms controlling the timing of human maturation might be shared across lineages.

## Abstract (web summary)

Neurons derived from stem cells are rapidly matured with a GENtoniK cocktail.

## Main

Recent advances in hPSC differentiation enable the derivation of a myriad of specific subtypes of neurons on demand. However, the application of this technology remains hampered by the slow maturation rates of human cells, resulting in prolonged culture periods for the emergence of disease-relevant phenotypes. Indeed, most neurological and psychiatric disorders manifest as impairments in postnatal or adult neuron functions such as synaptic connectivity^1^, dendritic arborization^2^ and electrophysiological function^3^. Therefore, developing strategies to accelerate the maturation of hPSC-derived neurons is critical to realize their full potential in modeling and treating neural diseases.

Multiple cell-extrinsic factors have been identified as contributors to neuron maturation, including glial cells^4^, network activity^5^ and neurotrophic factors^6^. However, within a given microenvironment, cell-intrinsic maturation rates appear dominant and determined by a species-specific molecular clock, which runs particularly slowly in human neurons^7,8^. For example, the maturation of hPSC-derived cortical neurons transplanted into the developing mouse brain follows human-specific timing, requiring 9 months to achieve mature, adult-like morphologies and spine function^9^. Similarly, the transplantation of mouse versus pig versus human midbrain dopamine neurons into the brain of Parkinsonian rats results in graft-induced functional rescue after 4 weeks, 3 months or 5 months, respectively, indicating that transplanted cells retain their intrinsic, species-specific, in vivo maturation timing rather than adopting the timing of the host^10^.

In the present study, we established a multi-phenotypic, image-based assay to monitor maturation in nearly pure populations of hPSC-derived, deep-layer cortical neuron cultures and applied it to screen 2,688 bioactive compounds for drivers of maturation. Among the screening hits, compounds targeting chromatin remodeling and calcium-dependent transcription were combined into a maturation cocktail that was effective across a broad range of maturation phenotypes and multiple cell types.

## Results

### High-content assay of neuron maturity

**Fig. 1:**
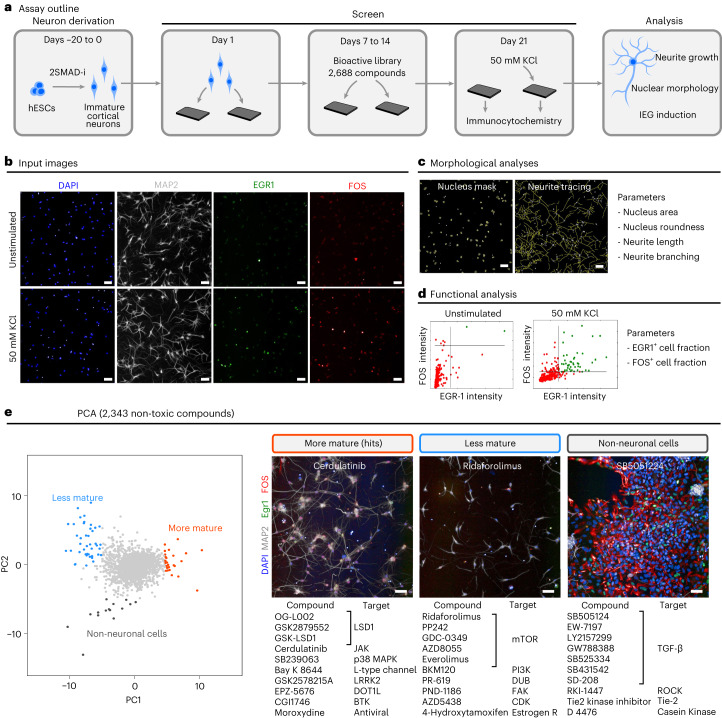
Chemical HCS for drivers of neuron maturation. **a**, Outline of screening protocol in hPSC-derived excitatory cortical neurons. 2SMAD-I, dual-SMAD inhibition. **b**, Example of input immunofluorescent images. Top, unstimulated neurons at day 21 post-plating. Bottom, neurons that received 50 mM of KCl 2 h before fixation. **c**, Automated analysis of neuron morphology. Left, nuclei detection mask from the DAPI channel. Right, automated neurite tracing from the MAP2 channel. **d**, Quantification of neuron excitability by applying an intensity threshold to FOS and EGR-1 channels within the nuclear mask. **e**, Left, PCA of screened compound library computed from six maturity parameters: nucleus area, nucleus roundness, total neurite length, number of neurite segments, FOS^+^ cell fraction and EGR-1^+^ cell fraction (*z*-scores averaged from *n* = 2 independent screens). Left, PCA plot of 2,343 nontoxic library compounds (out of 2,688 total compounds tested) with phenotypic clustering of maturation-enhancing (orange), maturation-inhibiting (blue) and non-neuronal proliferation-enhancing (gray) compounds. PC1 is primarily driven by the results from IEG induction and neurite growth, whereas PC2 is mainly driven by the nuclear size/roundness data. Right, representative screen images and ten representative hit compounds within each cluster. Scale bars, 50 μm. Source data

The phenotypic complexity of neurons makes single-readout assays unsuitable to fully capture maturation stages. Therefore, we used a multi-phenotype approach (via high-content screening (HCS)) to design an assay that monitors multiple features of neuronal maturation in parallel (Fig. 1a). Dendritic outgrowth is a widely used parameter of neuron maturity^11^ and can be monitored through automated tracing of microtubule-associated protein 2 (MAP2) immunostaining (Fig. 1b,c). Changes in nuclear size and morphology are also characteristic of neuron development and maturation^12^ and can be tracked via DAPI counterstaining (Fig. 1b,c). As an indirect measurement of neuronal function and excitability, we quantified the nuclear expression of immediate early gene (IEG) products FOS and early growth response (EGR)-1 after 2 h of KCl stimulation (Fig. 1b,d). IEGs are defined by their rapid induction without requiring new protein synthesis by stimuli that include sustained membrane depolarization in neurons^13^. In contrast to more traditional measures of neuronal activity such as calcium imaging and electrophysiology, IEG immunoreactivity is readily scalable as a readout for thousands of treatment conditions. However, IEGs can be triggered by stimuli other than neuronal activity, including growth factor signaling^14^ and cellular stress responses^15^. Therefore, to avoid direct activation of IEGs, we used transient compound treatment (days 7–14) and performed all measurements after removal of compounds, followed by culture in compound-free medium for an additional 7 d (days 14–21) before analysis (Fig. 1a). Furthermore, we recorded IEGs under both basal and KCl-stimulated conditions to specifically determine the depolarization-induced signal by subtracting the baseline from KCl-induced responses. Measuring maturation readouts only after compound withdrawal enabled the identification of hits that trigger a long-lasting ‘memory’ of a maturation stimulus even 1 week after compound withdrawal.

Although these readouts are pan-neuronal, and therefore appropriate across different neuronal lineages, we chose cortical neurons for the screen for both technical and biological reasons. Cortical neurons can be derived at high efficiency in the absence of expensive recombinant proteins and their even cell distribution in two-dimensional (2D) culture, free of clusters, makes them amenable to high-throughput imaging. They also represent a brain region that follows a particularly protracted timing of development and a region of great importance to human neurological disease. Our cortical neuron differentiation protocol yields enriched populations of post-mitotic deep-layer T-brain 1-positive (TBR1^+^) cells, which can be scaled, cryopreserved and directly thawed for use in large-scale assays (Supplementary Fig. 1a–e). To benchmark assay performance in mature cells, we employed primary embryonic rat cortical neurons, which quickly and reliably develop mature-like functionality in vitro^16^. At 14 d after plating, rat neurons displayed large and round nuclei (130 μm^2^, 0.93 roundness index), extensive neurite growth (>2,500 μm per neuron) and almost 100% of the neurons showed KCl-induced IEG responses (Supplementary Fig. 1f–j). In contrast, in hPSC-derived cortical neurons, these properties only very gradually emerged over a 50-d culture period and never reached the maturity of their rodent counterparts (Supplementary Fig. 1k–n). These results indicate that our multi-phenotypic assay reliably captures aspects of maturation in rat and hPSC-derived cortical neurons.

### Chemical screen for maturation enhancers

We next applied our maturity assay to screen a library of 2,688 bioactive compounds in hPSC-derived cortical neurons (Supplementary Fig. 2a). The library was applied at 5 μM and standard scores (*z*-scores) of duplicate screen runs were averaged for analysis. Viability was determined by quantifying intact nuclei and 325 toxic compounds with a viability *z*-score <−2 were excluded from further analysis (Supplementary Fig. 2b). For HCS hit selection, we applied principal component analysis (PCA) to six maturity *z*-scores to identify hit patterns for compounds, avoiding single threshold hit discrimination (Fig. 1e, left). The six parameters were: nucleus size and roundness, total neurite length and branching (number of segments per cell), and fractions of KCl-induced FOS^+^ and EGR-1^+^ cells. We identified three phenotypic clusters of compounds by PCA: maturation enhancers (hits); maturation suppressors, consisting mostly of inhibitors of the phosphoinositide 3-kinase (PI3K)/protein kinase B (AKT)/mechanistic target of rapamycin (mTOR) axis; and inducers of proliferation of a non-neuronal contaminant population, which were highly enriched for transcription growth factor (TGF)-β signaling inhibitors as well as inhibitors of ρ-associated protein kinase (ROCK) and other signaling pathways (Fig. 1e, right). We selected 32 compounds within the mature cluster (PC1 > 4) for validation. Although PCA identifies compounds with the greatest overall maturation effect, we reasoned that compounds with strong effects on single parameters could also be of interest. We therefore added the top five highest scoring compounds for each, total neurite length and double FOS^+^/EGR-1^+^ cells, excluding compounds already selected by PCA (Supplementary Fig. 3a). As single-parameter readouts are susceptible to false positives, we excluded drugs with known maturation-independent effects, such as the microtubule stabilizers docetaxel and paclitaxel. Neurite-only hits included inhibitors of Aurora kinase, in agreement with recent phenotypic screens targeting this phenotype^17,18^. Using such combined criteria, we selected 42 primary hits for follow-up studies (Supplementary Table 1).

**Fig. 2:**
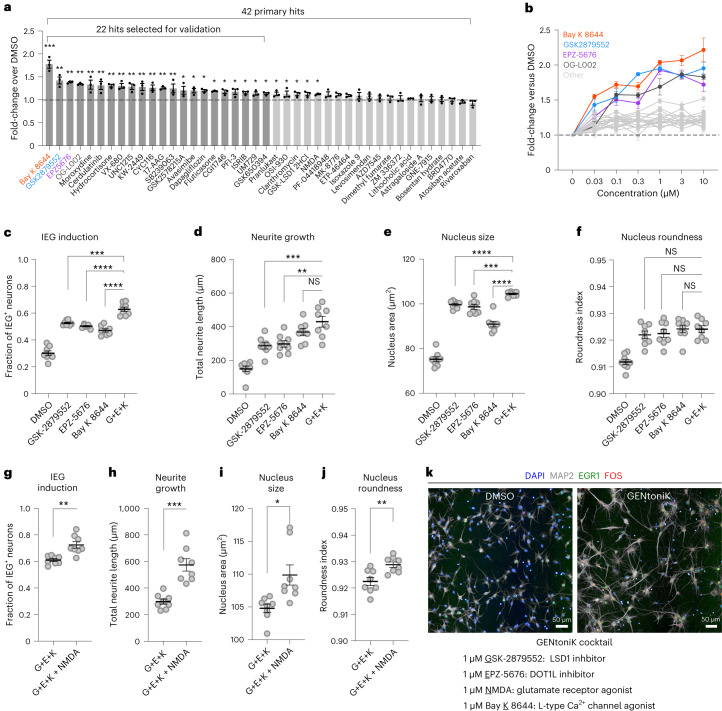
Validation and a combination of screen hits identify maturation-promoting cocktail GENtoniK. **a**, Ranking of primary hits by the mean of four maturity parameters (nucleus size and roundness, neurite length and KCl-induced double FOS^+^/EGR-1^+^ cells) normalized to DMSO (*n* = 3 microplate wells). The 22 top-ranked compounds were selected for validation. **b**, Dose–response validation of 22 screen hits comparing the mean of 4 maturity parameters normalized to DMSO (*n* = 15 microplate wells from 3 independent differentiations). **c**–**f**, Comparison of confirmed hits GSK2879552 (G), EPZ-5676 (E), Bay K 8644 (K) and a combination of the three (G + E + K) across maturity parameter IEG induction (**c**), neurite growth (**d**), nucleus size (**e**) and nucleus area (**f**) (*n* = 8 microplate wells from 2 independent experiments). **g**–**j**, Comparison of three-hit drug combination (G + E + K) to the same with the addition of NMDA across maturity parameter IEG induction (**g**), neurite growth (**h**), nucleus size (**i**) and nucleus roundness (**j**) (*n* = 8 microplate wells from 2 independent differentiations). **k**, Top, representative images of cortical neurons treated with DMSO or maturation-promoting cocktail GENtoniK. Bottom, formulation of GENtoniK. In **a** and **b**, Brown–Forsythe and Welch’s ANOVA with Dunnett’s T3 multiple-comparison test were used. In **c**–**j**, two-tailed Welch’s *t*-test was used; asterisks indicate statistical significance. Mean values are represented by a bar graph (**a**) or a line (**c**–**j**). Error bars represent s.e.m. Scale bars, 50 μm. Source data

To validate primary hits, the 42 compounds were subjected to the maturity assay in triplicate at the screening concentration (5 μM) and ranked by their effect on 4 maturity parameters: nucleus size and roundness, total neurite length and double positivity for KCl-induced FOS/EGR-1 cells (Supplementary Fig. 3b). The 22 compounds with the highest mean normalized score over dimethyl sulfoxide (DMSO) across all parameters underwent additional dose–response studies (Fig. 2a), resulting in the identification of four compounds with the most pronounced, dose-dependent effects on the mean maturation score (Fig. 2b). These compounds consisted of two inhibitors of lysine-specific demethylase 1 (LSD1/KDM1A), an inhibitor of disruptor of telomerase-like 1 (DOT1L) and an L-type calcium channel (LTCC) agonist. As the screen was run at a concentration susceptible to off-target effects, we conducted dose–curve experiments including independent compounds targeting DOT1L and LTCC, observing dose-dependent improvements across all maturation parameters (Supplementary Fig. 4). The identification of two additional LSD1 inhibitors as hits in the primary screen obviated this step for this target.

### Small-molecule cocktail promotes neuron maturity

LSD1 is a histone 3 demethylase at lysines 4 and 9, and a switch of specificity between these two substrates has been previously linked to neuron differentiation^19,20^. DOT1L is the sole methyltransferase targeting lysine 79 within the globular domain of histone 3 (ref. ^21^). LTCCs are involved in calcium-dependent transcription and play important roles in neuron development^22^. We reasoned that transcriptional induction by the LTCC agonist might act independently and further potentiate the effect of chromatin remodeling by epigenetic regulators such as LSD1 and DOT1L. Accordingly, we next sought to determine whether a combination of hits can further enhance neuron maturation. As two of the confirmed hits target LSD1, we decided to pursue only one of them, GSK2879552, for combinatorial experiments, because it displayed a stronger combined effect than OG-L002 (Fig. 2b). A combination of the three-hit compounds significantly increased IEG induction, neurite growth and nucleus size, but not nucleus roundness, compared with single-compound treatments (Fig. 2c–f).

In addition to LTCCs, calcium-dependent transcription is initiated through activation of *N*-methyl-d-aspartate (NMDA)-type glutamate receptors^23^, which have also been shown to participate in neuron maturation^24^. The compound NMDA itself was among the primary hits but, although significant, it was not among the 22 top hits in the single-agent validation study (Fig. 2a). Given its known role in activity-dependent transcription, we next tested whether the addition of NMDA could further enhance maturation in the presence of the above three-hit combination. We observed significant improvements across all maturity parameters (Fig. 2g–j) and nominated the resulting four drugs (GSK2879552, EPZ-5676, NMDA and Bay K 8644) as a maturation-promoting cocktail, naming it GENtoniK (Fig. 2k).

Dysregulation of both histone methylation and calcium signaling can be associated with toxicity in neurons. To determine potentially harmful effects of GENtoniK on neuronal cultures, we conducted viability and cellular stress assays in cortical neurons from WA09 human embryonic stem cell (hESC) and GM03348-induced hPSC (hiPSC) lines. Neither individual compounds nor the complete GENtoniK cocktail increased cell death compared with DMSO in a 21-d time-course analysis measuring plasma membrane integrity at the end-point (Supplementary Fig. 5a,b). In fact, a resazurin-based assay resulted in a slightly improved viability (Supplementary Fig. 5c), possibly owing to higher respiratory rates of treated neurons caused by increased surface area and metabolism. As a readout of double-strand DNA breaks, we quantified nuclear foci containing phosphorylated ATM (serine/threonine kinase), observing no difference between DMSO and GENtoniK neurons (Supplementary Fig. 6a, at 24 h post-treatment, and Supplementary Fig. 6c, at 24 h and 7 d post-treatment), but a dramatic increase in those treated with the radiomimetic drug bleomycin as a positive control. To assess potential copy number aberrations (CNAs) induced by GENtoniK treatment, we conducted shallow whole-genome sequencing (WGS), observing no difference in copy number profiles of GENtoniK- and DMSO-treated neurons (Supplementary Fig. 6c). GENtoniK also did not cause obvious aberrations in chromatin nuclear localization, as revealed by staining for markers of heterochromatin and active chromatin H3K9me3 and H3K9ac (Supplementary Fig. 6d). Similarly, there was no loss of H3K9me3 intensity or percentage positive cells by flow cytometry upon GENtoniK treatment (Supplementary Fig. 6e–h).

### GENtoniK promotes functional neuron maturation

**Fig. 3:**
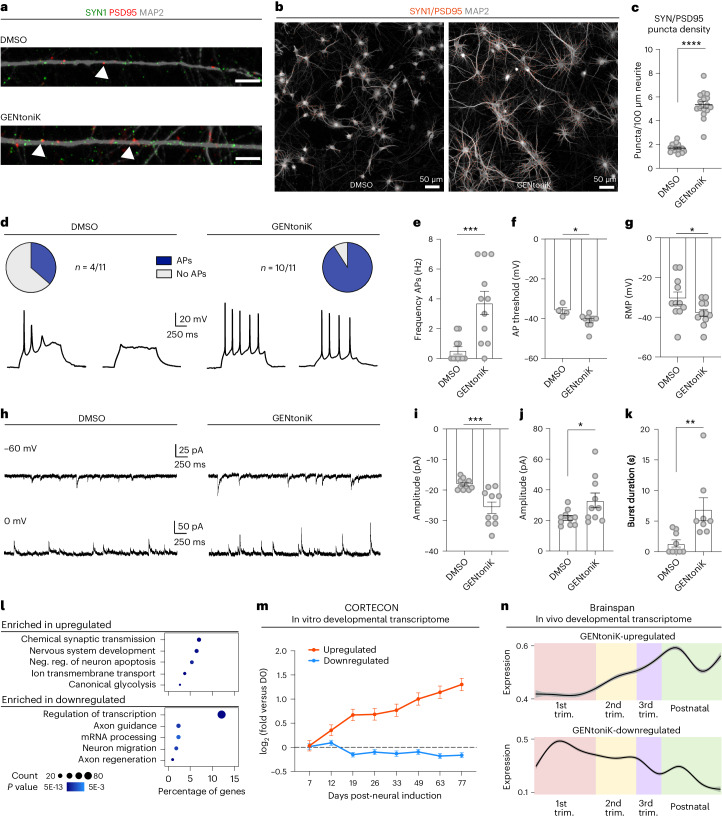
Validation of small-molecule maturation strategy with screen-independent readouts. **a**, Structured illumination (apotome) imaging of pre- and postsynaptic markers SYN1 and PSD95 in hPSC neurons, with marker apposition highlighted by arrows. Scale bars, 10 μm. **b**, Representative images of synaptic markers in hPSC neurons. The orange dots represent instances of SYN1 and PSD95 apposition. Scale bars, 50 μm. **c**, GENtoniK increasing density of double-positive puncta per neurite length (*n* = 16 wells from *n* = 2 independent differentiations). **d**–**g**, GENtoniK promoting excitability and mature resting properties of hPSC neurons. More than 90% treated neurons fired evoked APs compared with <40% of DMSO controls (**d**): representative traces shown for each group: quantification of AP frequency (*n* = 11 cells per group) (**e**); AP threshold (*n* = 4 for DMSO and *n* = 10 for GENtoniK) (**f**); and resting membrane potential (RMP) (*n* = 11 per group) (**g**). **h**–**k**, GENtoniK increasing sEPSCs amplitude and burst duration in hPSC neurons: representative traces of sEPSCs (**h**); quantification of sEPSC amplitude recorded at –60 mV (*n* = 11 cells for DMSO and *n* = 10 for GENtoniK) (**i**); sEPSC amplitude at 0 mV (*n* = 10 per group) receptors (**j**); and sEPSC duration (*n* = 9 for DMSO and *n* = 8 for GENtoniK) (**k**). **l**, GO analysis showing enrichment for mature neuron function in genes upregulated by GENtoniK and enrichment for immature function and transcriptional regulation in genes downregulated by GENtoniK. The significance was determined using Fisher’s exact test. **m**, Genes upregulated by GENtoniK displaying increasing average expression with increased days in culture in the CORTECON transcriptome of in vitro cortical development (https://cortecon.neuralsci.org), whereas genes downregulated by GENtoniK remained constant (*n* = 3 independent differentiations, data shown as mean values ± s.d.). **n**, In the BrainSpan Atlas of the Developing Human Brain (https://www.brainspan.org), genes upregulated by GENtoniK displaying an average expression that increased from early development to gestation and after birth (top). trim., trimester. Genes downregulated display higher average expression during early development and decrease over time (bottom). The black line represents smoothed mean curves with bands representing confidence intervals. In **c**, **e**–**g** and **i**–**k**, two-tailed Welch’s *t*-test was used; asterisks indicate statistical significance. Mean values are represented by a black line (**c**) or a bar graph (**e**–**g** and **i**–**k**). Error bars represent s.e.m. Source data

We next validated GENtoniK on additional maturation phenotypes, independent of those assayed during primary screening. Establishing independent functional readouts was particularly important, because three of the proteins targeted by the cocktail have been reported to directly participate in IEG induction in neurons^25–27^. The formation of chemical synapses is a critical step in neuronal development, which again occurs in a protracted manner in the human cortex^28^. We used immunofluorescent staining on day-35 cortical neurons to assess the effect of GENtoniK on synaptogenesis. We confirmed an increase in overall neurite length and branching assessed by MAP2 staining, which is enriched in dendrites (Supplementary Fig. 7a). Notably, GENtoniK-treated neurons showed increased density of pre- and postsynaptic markers SYN1 (Synapsin 1) and PSD95 normalized by neurite length (Supplementary Fig. 7b–d). Structured illumination optical sectioning revealed the apposition of the two markers, indicating synaptic assembly of pre- and postsynaptic elements (Fig. 3a). To quantify the density of assembled synapses per neurite length, we employed HCS, observing a marked increase in GENtoniK-treated neurons across multiple hPSC lines (Fig. 3b,c and Supplementary Fig. 14).

Neurite quantification in our screen relied on the dendrite-specific marker MAP2. We next asked whether GENtoniK also enhances axonal maturation. Formation of the axon initial segment (AIS) has been used as a maturation marker in cortical neurons^29^. However, we observed that, in our neurons, an ANK3^+^ AIS is present even in untreated neurons at 21 d from plating, suggesting that it is not a limiting factor in maturation (Supplementary Fig. 8a). Expression of the pathologically relevant, four-repeat tau isoform is a feature of axonal maturity and important for disease modeling whereas the shorter three-repeat isoform is expressed during early fetal development^30^. Western blotting revealed increased levels of four-repeat tau in GENtoniK-treated neurons when normalized to both total tau and glyceraldehyde 3-phosphate dehydrogenase levels (Supplementary Fig. 8b–d).

Intrinsic electrophysiological features, such as passive membrane properties and the ability to fire action potentials (APs), are important functional indicators of neuronal maturation^31^. To assess the effect of the drug cocktail on membrane properties and excitability, we performed whole-cell, patch-clamp recordings in cortical neurons at day 28 from plating. Similar to the IEG studies, treatment was withdrawn 7 d before recordings to ensure that differences were maturation mediated and not a direct effect of NMDA or Bay K 8644. Over 90% of GENtoniK-treated neurons displayed evoked APs compared with <40% of control neurons (Fig. 3d). Among AP-firing neurons, those treated with GENtoniK displayed higher firing frequencies (Fig. 3e) and lower AP thresholds (Fig. 3f). Despite lower resting membrane potential in treated neurons (Fig. 3g), their values were still distant from the physiological range of −60 mV to −70 mV reported for the cortex in vivo^32^. These results indicate that GENtoniK significantly promotes excitability, but that additional, including extrinsic, factors may be required to achieve more mature resting membrane properties. Finally, to probe for functional consequences of enhanced synaptic marker expression in GENtoniK-treated neurons (Fig. 3a–d), we recorded spontaneous excitatory postsynaptic currents (sEPSCs) and found that treated neurons displayed increased sEPSC amplitudes when recorded at either –60 mV or at 0 mV (Fig. 3h–j). GENtoniK-treated neurons displayed more pronounced, synchronized bursts of sEPSCs (Fig. 3k) as further evidence of enhanced synaptic maturation.

### GENtoniK induces immature to mature shift in transcription

We next conducted RNA sequencing (RNA-seq) to assess global changes in gene expression induced by GENtoniK. In accordance with a dual effect of the cocktail on chromatin state and calcium influx, we treated hPSC cortical neurons with either of the two epigenetic factors, the two compounds affecting calcium signaling or the full GENtoniK cocktail (Supplementary Fig. 9a). Genes differentially expressed in GENtoniK were similarly regulated by treatment with the two epigenetic drugs, but to a lesser magnitude, which is consistent with the hypothesis that calcium influx potentiates transcriptional changes facilitated by chromatin remodeling (Supplementary Fig. 9b–d). Although both calcium signaling modulators were identified as maturation enhancers in our protein-based screen, their combined effect on gene expression was modest 7 d after treatment withdrawal (Supplementary Fig. 9b).

Gene ontology (GO) analyses of transcripts downregulated by GENtoniK revealed enrichment in immature, early, post-mitotic neuron functions, including migration and axon guidance, as well as transcriptional regulation (Fig. 3l and Supplementary Fig. 9e). Upregulated genes were enriched for transcripts related to mature neuron function, including chemical synaptic transmission and transmembrane ion transport (Fig. 3l and Supplementary Fig. 9f). Although previous studies indicate a switch from glycolytic to oxidative metabolism in maturing neurons^33,34^, we observed enrichment in both glycolysis and oxidative phosphorylation, as well as fatty acid metabolism in treated cells (Supplementary Fig. 10).

To match the transcriptional data with temporal changes in gene expression during cortical neuron development in vitro, we plotted differentially expressed genes against the CORTECON dataset^35^. Genes upregulated by GENtoniK showed a time-dependent increase in expression on extended in vitro culture (Fig. 3m), as expected for maturation-related transcripts. However, the CORTECON data do not allow precise staging of GENtoniK-treated neurons. To match in vitro transcriptional data with in vivo changes in gene expression, we mapped differentially expressed genes against the BrainSpan Transcriptome database of the Developing Human Brain^36^. Most genes upregulated by GENtoniK showed an increase in gene expression from early to late gestation (Fig. 3n, top). In contrast, genes downregulated by the treatment were more highly expressed during early embryonic brain development, with decreasing levels toward birth and postnatal stages (Fig. 3n, bottom). There were no changes in markers of neuronal and glial subtype identity in GENtoniK-treated neurons (Supplementary Fig. 11a).

Recent studies reported transcriptional signatures of cell stress that may interfere with the development and maturation of cortical organoids^37^. Gene expression analysis in GENtoniK-treated neurons did not reveal any enrichment for genes associated with the integrated stress response (Supplementary Fig. 11b). Finally, neuronal maturation has been linked to developmental switches from fetal to adult variants of specific transcripts. One such example is the switch from NMDAR subunits GRIN2B to GRIN2A^38^. Our RNA-seq results revealed significant decreases in the fetal GRIN2B subunit in GENtoniK-treated neurons from three of four independent hPSC lines. However, we did not observe a significant upregulation of the adult GRIN2A subunit (Supplementary Fig. 11c), suggesting that longer-term GENtoniK treatment, more extended culture periods or additional factors may be required to trigger a complete switch to adult-like NMDAR subunits.

We next performed CUT&RUN chromatin profiling on histone marks downstream of the epigenetic factors targeted by the cocktail. Although LSD1 can switch its substrate to H3K9 in the mature neuron-specific variant, we focused on its canonical target H3K4, reasoning that maturation-enhancing inhibition probably targets the immature form. H3K4me2 was widespread in the genome, with the highest enrichment in the promoter region and near the transcription start site (Supplementary Fig. 12a). In contrast, H3K79me2 was enriched at a much smaller subset of genes, where it extended into the transcribed region (Supplementary Fig. 12b). Both H3K4 and H3K79 2-methylation were more highly enriched at GENtoniK-downregulated versus GENtoniK-upregulated genes (Supplementary Fig. 12c). Genes associated with H3K79 peaks showed near-identical ontology enrichment to those downregulated with GENtoniK by RNA-seq, including terms for neuron migration, chromatin modification and RNA-processing gene categories (Supplementary Fig. 12d–g). Chromatin-regulating genes associated with H3K79me2 peaks include the GENtoniK target LSD1 (Supplementary Fig. 12f), whereas messenger RNA-processing genes with H3K79me2 peaks, such as *NOVA2* and *CELF1* (Supplementary Fig. 12g), are known to participate in cortical neuron development^39,40^. These results indicate that H3K79 methylation may play a role in maintaining immature gene expression programs and that loss of this mark might facilitate neuronal maturation in GENtoniK-treated cells.

### GENtoniK enhances maturation across neuronal culture systems

We next tested the efficacy of GENtoniK across additional hPSC lines and hPSC-derived cell types. As our primary screen was carried out in a female hESC line H9 (WA09), we first replicated key maturation readouts in cortical neurons derived from male and female induced PSC (iPSC) lines. We confirmed the effect of GENtoniK on both IEG induction and neurite outgrowth across iPSC lines from five donors (Supplementary Fig. 13a–e). Functional maturation was assessed by validating changes in synaptic markers and changes in maturation-associated gene expression. We observed a robust increase in total number of assembled synapses relative to DMSO controls in GENtoniK-treated neurons across iPSC lines (Supplementary Fig. 14a–c). Finally, bulk RNA-seq analysis of iPSC neurons showed an upregulation in gene expression pathways associated with maturation, including chemical synaptic transmission, and downregulation of genes associated with immature post-mitotic neurons, including axon guidance (Supplementary Fig. 15a,c,e). Comparison with the Brainspan Human Developmental Atlas (Brainspan.com) showed that genes upregulated by GENtoniK were enriched in postnatally associated transcripts, whereas genes downregulated by GENtoniK were primarily associated with early prenatal development (Supplementary Fig. 15b,d,f). Collectively, these results confirm that GENtoniK promotes the maturation of hPSC-derived neurons, independent of biological sex or hESC versus iPSC origin.

Alternative maturation strategies are routinely employed in neuronal cultures, including the addition of trophic factors such as brain-derived neurotrophic factor (BDNF), and the use of culture medium with more physiological levels of glucose and ion concentrations (BrainPhys)^41^. We conducted time-course experiments to assess the efficacy and compatibility of GENtoniK with existing maturation approaches. GENtoniK in standard neurobasal medium (without neurotrophic factors) robustly induced several neuronal maturation parameters at levels above control or BrainPhys plus BDNF conditions. Treatment with GENtoniK in combination with BrainPhys and neurotrophic factors showed an additional, albeit modest, increase in maturation (Supplementary Fig. 16). More detailed electrophysiological and transcriptional studies will be required to fully define the complementary and synergistic effect of GENtoniK and BrainPhys on neuronal maturation.

**Fig. 4:**
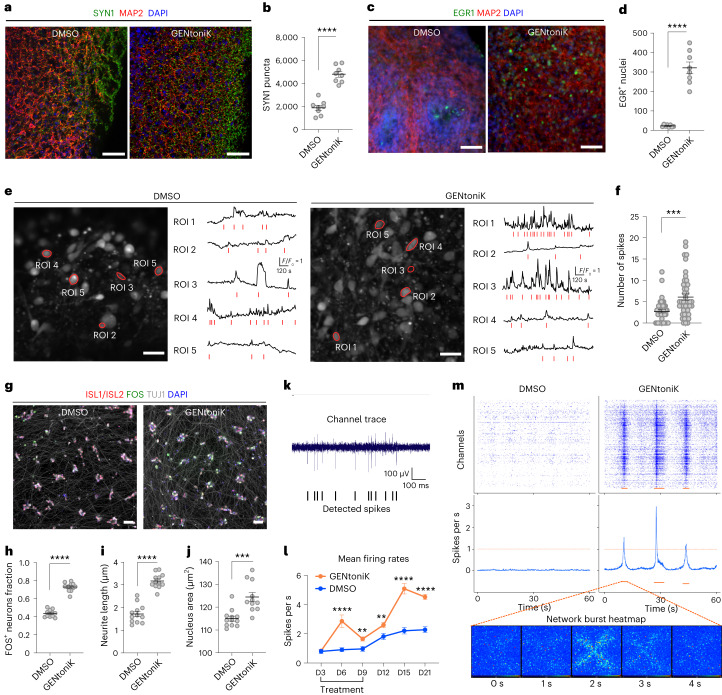
Validation of maturation strategy across hPSC-derived neuronal cultures. **a**–**f**, GENtoniK treatment inducing synaptogenesis and spontaneous activity in cortical organoids. **a**, Representative images of immunofluorescent staining for SYN1 and MAP2 in day-60 organoids. **b**, Quantification of total SYN1 puncta per field (*n* = 8 cryosections randomly sampled from *n* = 20 organoids). **c**, Representative images of immunofluorescence staining for EGR-1 and MAP2 in unstimulated day-60 organoids. **d**, Quantification of EGR-1^+^ cells per field (*n* = 8 cryosections randomly sampled from *n* = 20 organoids). **e**, Representative confocal images with ROI traces of calcium imaging with Fluo-4 AM in whole day-60 cortical organoids that received treatment with DMSO (left) or GENtoniK (right). Traces show a 20-min time course captured at 0.2 ps. The red lines represent counted spikes. **f**, Quantification of total spikes per ROI in 20-min recordings of Fluo-4 AM calcium transients (*n* = 45 DMSO ROIs and 47 GENtoniK ROIs from 4 organoids per condition). **g**–**m**, GENtoniK promoting maturation of hPSC-derived SMNs. **g**, Representative high-content maturation assay images of ISL1/2^+^ SMNs (day 40 of hPSC differentiation). **h**–**j**, Quantification showing GENtoniK-improved KCl-induction of FOS^+^ cells (**h**), total neurite length (*n* = 12 for both conditions) (**i**) and nucleus area (*n* = 12 for both conditions) (**j**) in SMNs (*n* = 12 for DMSO and *n* = 11 for GENtoniK). **k**–**m**, GENtoniK treatment increasing firing rates and inducing spontaneous bursting activity on SMNs plated on high-density multielectrode arrays. **k**, Sample single-channel trace of GENtoniK-treated SMNs illustrating spike detection. **l**, Time-course analysis of average firing rates in SMNs plated on high-density microelectrode arrays (HD-MEAs), calculated from 60 s of activity in the 1/64th most active electrodes (*n* = 128 electrodes from 2-MEA probes). **m**, Representative 60-s spike rastergrams (top) and average firing rates (middle) of SMNs plated on HD-MEAs. Only GENtoniK-treated SMNs displayed spontaneous bursting events (orange bars). Bottom, array heatmap of a 4-s bursting event. In **b**, **d**, **f**, **h**–**j** and **l**, two-tailed Welch’s *t*-test was used. Asterisks indicate statistical significance. Mean values are represented by a black line. Errors represent s.e.m. Scale bars, 50 μm. Source data

Self-organizing 3D culture systems such as neural organoids have become a widely used model system to study human brain development and disease. Similar to 2D culture systems, human 3D organoids are subject to slow maturation rates^42^. We observed that forebrain organoids treated with GENtoniK, from day 15 to day 50 of derivation, displayed an increased density of SYN1 puncta (Fig. 4a,b), decreased expression of immature neuron marker DCX (Supplementary Fig. 17a) and increased number of cells with nuclear expression of EGR-1 and FOS (Fig. 4c,d and Supplementary Fig. 17a) at day 60 without inducing obvious changes in the cortical layer identity (Supplementary Fig. 17b). For these studies, organoids were not subjected to KCl stimulation before IEG immunostaining, thus indicating higher levels of spontaneous activity after GENtoniK treatment. To confirm this effect functionally, we next conducted calcium imaging in intact organoids loaded with the calcium indicator Fluo-4 AM. In agreement with the IEG results, regions of interest (ROIs) within GENtoniK-treated organoids displayed an increased number of spontaneous calcium spikes (Fig. 4e,f and Supplementary Videos 1 and 2).

We next addressed whether the treatment could drive the maturation of hPSC-derived neurons outside the forebrain by deriving spinal motoneurons (SMNs). ISL1^+^ SMNs treated with GENtoniK displayed a highly significant increase across all the maturity parameters tested (Fig. 4g–j). We observed that SMNs exhibit robust levels of spontaneous activity when cultured on high-density multielectrode arrays (Fig. 4k). In a time-course experiment, average firing rates were increased modestly in the presence of the drug cocktail (possibly via direct ion channel activation effect). In contrast, a more pronounced effect was observed starting 6 d after treatment withdrawal, indicating that the treatment triggered a long-lasting maturation effect (Fig. 4l). Notably, only SMNs pretreated with GENtoniK exhibited highly synchronous bursts of activity in the 0.8- to 0.6-Hz range (Fig. 4m and Supplementary Video 3), reminiscent of central pattern generator activity in the embryonic spinal cord^43^.

### GENtoniK enhances cell function in non-neuronal lineages

Slow maturation rates of hPSC-derived cells are a common problem across lineages beyond neurons. To assess the potential of GENtoniK in other cell types, we next tested neural crest-derived melanocytes, which produce the pigment melanin in a differentiation/maturation-dependent manner. The production and secretion of melanin from melanocytes is responsible for human skin and hair color, and hPSCs/melanocytes have been used to model various pigmentation disorders^44^. Using our established differentiation protocol, treatment of hPSC-derived melanocytes with GENtoniK, starting at day 11, induced a dramatic increase in pigmentation at day 33 of differentiation versus untreated melanocytes (Supplementary Fig. 18).

Finally, we tested GENtoniK on a cell type derived from a different germ layer, hPSC-derived. insulin-secreting, pancreatic β-like cells. These cells arise from definitive endoderm and are of great interest for developing cell-based treatments for type 1 diabetes^45^. Although many protocols have been reported, one limitation is the generation of a subset of glucagon-positive (GCG^+^), insulin-positive (INS^+^) polyhormonal cells^46^. Flow cytometry analysis revealed that GENtoniK treatment decreased the number of GCG^+^ cells among INS^+^ cells (Supplementary Fig. 19b,c). The β-like cells treated with GENtoniK from day 20 to day 27 of differentiation displayed evidence of improved maturation, including increased total insulin content, fraction of insulin granules and KCl-induced insulin secretion at day 29 (Supplementary Fig. 19d–i). These results suggest that GENtoniK can trigger aspects of cellular function, differentiation or maturation also in non-neural lineages.

## Discussion

We present a combined chemical strategy aimed at promoting the maturation of human stem cell-derived neurons, which we obtained by combining hits from a high-content small-molecule screen. Applying a multiparameter readout enabled us to identify compounds that effectively drive neuronal maturation rather than simply promoting individual features such as neurite outgrowth^47,48^. PCA of the screen results yielded three phenotypic clusters of compounds that either promoted or inhibited neuronal maturation and compounds that triggered the growth of non-neural contaminants. The enrichment of mTOR and PI3K regulators among maturation inhibitors concurs with recent findings proposing mTOR activation as a driver of interneuron maturation^49^. An unexpected finding was the identification of TGF-β and ROCK inhibitors as compounds promoting a ‘flat cell’ non-neuronal fate, which is a known contaminant of neural differentiations and thought to represent a neural crest^50^ or fibroblast-like^51^ mesenchymal cell lineage. Both TGF-β and ROCK inhibitors are commonly used in neural differentiation protocols, but our results indicate that they may promote undesired cell types if used at later differentiation stages.

A central finding of our study was the presence of an epigenetic program in immature neurons that prevents rapid maturation of human neurons. We hypothesized that GENtoniK acts in a two-pronged manner. The epigenetic probes GSK2879552 and EPZ-5676 induce a shift in chromatin accessibility from an immature (migration, axon guidance) to a mature transcriptional program (synaptic transmission, ion channel subunits). We further speculate that those changes in chromatin state facilitate NMDA- and Bay K 8644-mediated activation of calcium-dependent transcription^23^ as an additional driver of maturation.

We identified several inhibitors of LSD1 in our primary screen. LSD1 has been reported to regulate differentiation and maturation in olfactory^20^ and cortical neurons^52,53^, specifically as a member of the CoREST repressor complex. In addition to its roles in development, LSD1 participates in a myriad of functions in a highly context- and complex-specific manner, highlighting the importance of limiting the time of treatment to minimize off-target effects. Alternatively, functional specificity could be mediated by targeting individual complexes. Although a CoREST-specific probe has been developed^54^, in our hands this probe was highly toxic, preventing an assessment of any direct effects on neuronal maturation. DOT1L can modulate cell-cycle exit during neuronal differentiation^55^, but its role in regulating post-mitotic maturation has not been studied. Our chromatin-profiling data in immature neurons indicate that the DOT1L substrate H3K79me2 may be involved in controlling the accessibility of other transcriptional regulators including LSD1, making it an intriguing candidate as a potential master regulator of gene expression during development. In agreement with this observation, H3K79me2 levels globally increase alongside chromatin condensation during neuronal differentiation^56^, suggesting that it may participate in establishing an ‘epigenetic barrier’ at the transition from pluripotent cells to neural progenitors and immature neurons—a barrier then retained in human neurons for protracted periods during neuronal maturation. Given that its valence appears to be primarily determined by the rate of nucleosome turnover^57^, H3K79 methylation appears to be a plausible timekeeper in development.

An important issue to address is whether transient treatment with GENtoniK (or any other future methods of accelerating neuronal maturation) will affect the final molecular and functional properties of mature neurons. It is possible that accelerating maturation could trigger abnormal morphological and functional properties or induce properties that mimic those of neurons found in more rapidly maturing species. Therefore, detailed, single-cell transcriptional, epigenetic and morphological studies will be required to address this point in the future. The development of human atlases that capture single-cell-based maturation profiles of specific neuron subtypes will be critical for such comparisons. Furthermore, detailed mechanistic studies using inducible and reversible genetic perturbations will be required to determine the relative contribution of each pathway in promoting ‘on-target’ and potential ‘off-target’ features of maturation.

Although GENtoniK clearly triggers multiple aspects of neuronal maturation, there is ample room for further improvements, because neurons still do not acquire fully adult-like properties. Therefore, it will be important to assess whether the combination of GENtoniK with extrinsic strategies, such as co-culture with glial cells or conditioned medium, yields human neurons more fully matching adult-like function. Given a strong maturation effect observed in our motor neuron paradigm, even after GENtoniK treatment before post-mitotic neuron exit, future studies should test whether early treatment can prime neuronal precursors toward accelerated maturation, even in the absence of GENtoniK treatment at the post-mitotic stage.

We demonstrate that the same chemical strategy promotes aspects of functional maturation and/or differentiation in non-neuronal cells. However, in-depth studies will be required to better define the maturation process in those cell types and to fine-tune the cocktail for driving maturation. For example, although NMDARs and voltage-gated calcium channels have demonstrated functions in melanocytes and pancreatic β-like cells^58–61^, their activation might be dispensable in other cells, where alternative factors such as hormones might be required instead. Similarly, additional, alternative epigenetic regulators may contribute to maturation rates in other cell types and organ systems to assure appropriate tissue- and species-specific timing. Recent studies have shown that differences in the rate of biochemical reactions including protein synthesis and degradation correlate with species-specific differences in somite and spinal cord development^62,63^. However, further studies are needed to demonstrate a causal relationship and to elucidate whether those mechanisms apply to later developmental stages such as neuronal maturation. GENtoniK provides a simple and probable complementary strategy to accelerate maturation timing in neuronal and some non-neural cell types.

## Methods

Experimental details are summarized in Supplementary Table 2.

### Cell culture

#### Human PSCs

PSCs, both embryonic and induced, were maintained in Essential 8 medium (Thermo Fisher Scientific) on vitronectin-coated plates as previously described^64^. All stem cell work was conducted according to protocols approved by the Tri-Institutional Stem Cell Initiative Embryonic Stem Cell Research Oversight Committee (Tri-SCI ESCRO).

#### Human PSC-derived excitatory cortical neurons

Cortical neurons were generated using a protocol based on the previously described dual-SMAD inhibition paradigm^65^. Briefly, hESCs were dissociated into single cells with Accutase and seeded at 250,000 cm^−2^ on to Matrigel-coated plates in Essential 8 medium with 10 μM Y-27632. During days 1–10 of the protocol, medium consisted of Essential 6 (Thermo Fisher Scientific) with 10 μM SB431542 (Tocris) and 100 nM LDN193189 (Stemgent). Wnt inhibitor XAV-939 at 2 μM was included from day 1 to day 3 to improve anterior patterning^66^. On days 11–20, medium consisted of N-2-supplemented Dulbecco’s modified Eagle’s medium and Ham’s F-12 nutrient mixture (DMEM/F12; Thermo Fisher Scientific). Cells received daily medium exchanges throughout the differentiation. On day 20 cells were dissociated in Accutase for 30 min and plated on poly(l-ornithine) and laminin-coated (PLO/Lam) plates, in low-glucose (5 mM) Neurobasal A medium supplemented with 2% B27 and 1% GlutaMAX (Thermo Fisher Scientific) or cryopreserved in STEM-CELLBANKER solution (Amsbio). Neurons received medium exchanges twice a week. During the first 7 d after plating, medium was supplemented with notch-inhibitor DAPT (*N*-(*N*-(3,5-difluorophenacetyl)-l-alanyl)-*s*-phenylglycinet-butyl ester, a γ-secretase inhibitor) at 10 μM (ref. ^67^). Long-term cultures were maintained with BDNF (10 ng ml^−1^ of PeproTech), glial cell line-derived neurotrophic factor (GDNF; 10 ng ml^−1^, R&D Biosystems), dibutyryl cAMP (100 μM, Sigma-Aldrich) and ascorbic acid (100 μM, Sigma-Aldrich). However, owing to the activation of immediate early genes by BDNF and cAMP, the initial screen and validation experiments involving IEG induction were done in the absence of those factors.

#### Primary embryonic rat cortical neurons

Cells were obtained from Thermo Fisher Scientific, thawed following the vendor’s instructions and maintained in the same manner as hPSC cortical neurons.

#### SMNs

Motoneuron derivation was adapted from a previously described protocol^68^. In brief, Accutase-dissociated hESCs were seeded at 600,000 cm^−2^ on to Geltrex-coated plates and underwent dual-SMAD inhibition in the presence of CHIR99021 and Smoothened agonist. On day 11, spinal progenitors were collected and plated on PDL/Lam/FN plates and maintained in N-2/B27 medium containing Smoothened agonist, retinoic acid, BDNF, GDNF, ciliary neurotrophic factor (CTNF) and DAPT. On day 24, SMNs were re-plated on PDL/Lam/FN and maintained in Neurobasal medium supplemented with 2% B27, ascorbic acid, retinoic acid, BDNF, GDNF and CTNF. Treatment with GENtoniK or DMSO was initiated the day after re-plating.

#### Dorsal forebrain organoids

Organoids were derived from a previously reported protocol^69^. Briefly, 10,000 EDTA-dissociated hPSCs were plated per well of a 96-well, V-bottomed, low-attachment plate (S-bio). Cells were allowed to self-aggregate in hPSC growth medium overnight. From day 1 to day 8, medium was changed every 2 d with Essential 6 medium supplemented with 10 μM SB431542, 100 nM LDN193189 and 2 μM XAV-939. On day 8, the medium was switched to organoid growth medium consisting of a 50:50 mixture of Neurobasal and DMEM/F12 medium with 1% NeuroBrew 21 (Miltenyi), 0.5% N2, 1% GlutaMAX, 0.5% minimal essential medium (MEM) nonessential amino acid (NEAA) solution, 0.1% 2-mercaptoethanol and 1 μM recombinant human insulin (Sigma-Aldrich). On day 14 organoids were transferred to 10-cm dishes at roughly 20 organoids per dish and placed on an orbital shaker set to gentle motion to prevent organoid fusion.

#### Melanocytes

Melanocyte differentiation was executed as previously reported^70^. In brief, the day before differentiation, hPSCs were plated on Matrigel at 200,000 cells cm^−2^ in Essential 8 medium with 10 μM Y-27632. From day 0 to day 11 of the protocol, cells received daily exchanges of Essential 6 medium containing: 1 ng ml^−1^ of bone morphogenetic protein (BMP)4, 10 μM SB431542 and 600 nM CHIR99021 (days 0–2); 10 μM SB431542 and 1.5 μM CHIR99021 (days 2–4); 1.5 μM CHIR99021 (days 4–6); and 1.5 μM CHIR99021, 5 ng ml^−1^ of BMP4 and 100 nM EDN3 (days 6–11). On day 11, cells were dissociated into single cells with Accutase for 20 min and cKIT^+^ melanoblasts were sorted using an allophycocyanin-conjugated antibody (Thermo Fisher Scientific) on a BD-FACS Aria cell sorter at the Memorial Sloan–Kettering Cancer Center (MSKCC) Flow Cytometry Core Facility, and re-plated on to dried PO/Lam/FN dishes. Cells were fed with melanocyte medium (Neurobasal medium supplemented with: 50 ng ml^−1^ of stem cell factor, 500 μM cAMP, 10 ng ml^−1^ of fibroblast growth factor (FGF)2, 3 μM CHIR99021, 25 ng ml^−1^ of BMP4, 100 nM EDN3, 1 mM l-glutamine, 0.1 mM MEM NEAA solution, 2% B27 and 2% N-2) every 2–3 d and passaged using Accutase at a ratio of 1:4 once a week.

#### Pancreatic β-cells

β-Cell differentiation was performed using *INS*^*GFP/W*^ MEL-1 cells. Cells were maintained on Matrigel-coated plates in StemFlex medium (Thermo Fisher Scientific) at 37 °C with 5% CO_2_. MEL-1 cells were differentiated using a previously reported strategy^71^. Briefly, on day 0, cells were exposed to basal Roswell Park Memorial Institute (RPMI) 1640 medium (Corning) supplemented with 1× GlutaMAX (Thermo Fisher Scientific), 50 μg ml^−1^ of Normocin, 100 ng ml^−1^ of activin A (R&D systems) and 3 μM CHIR99021 (Cayman Chemical) for 24 h. The medium was changed on day 2 to basal RPMI 1640 medium supplemented with 1× GlutaMAX, 50 μg ml^−1^ of Normocin, 0.2% fetal bovine serum (FBS; Corning) and 100 ng ml^−1^ of activin A for 2 d. On day 4, the resulting definitive endoderm cells were cultured in MCDB131 medium supplemented with 1.5 g l^−1^ of sodium bicarbonate, 1× GlutaMAX, 10 mM glucose, 2% bovine serum albumin (BSA), 50 ng ml^−1^ of FGF7 and 0.25 mM ascorbic acid for 2 d. On day 6, the cells were differentiated in MCDB131 medium supplemented with 2.5 g l^−1^ of sodium bicarbonate, 1× GlutaMAX, 10 mM glucose, 2% BSA, 0.25 mM ascorbic acid, 2 μM retinoic acid, 0.25 μM SANT1, 50 ng ml^−1^ of FGF7, 200 nM tetraphenyl butadiene (TPB), 200 nM LDN193189 and 0.5× insulin–transferrin–selenium–ethanolamine (ITS-X) supplement for 2 d to pancreatic progenitor stage 1 cells. On day 8, the cells were directed to pancreatic progenitor stage 2 cells in MCDB131 medium supplemented with 2.5 g l^−1^ of sodium bicarbonate, 1× GlutaMAX, 10 mM glucose, 2% BSA, 0.25 mM ascorbic acid, 0.2 μM retinoic acid, 0.25 μM SANT1, 2 ng ml^−1^ of FGF7, 100 nM TPB, 400 nM LDN193189 and 0.5× ITS-X supplement for 3 d. On day 11, the cells were directed to insulin-expressing cells in MCDB131 medium supplemented with 1.5 g l^−1^ of sodium bicarbonate, 1× GlutaMAX, 20 mM glucose, 2% BSA, 0.1 μM retinoic acid, 0.25 μM SANT1, 200 nM LDN193189, 1 μM triiodothyronine (T_3_), 10 μM ALKi5, 10 μM zinc sulfate, 10 μg ml^−1^ of heparin and 0.5× ITS-X for 3 d. On day 14, the cells for static or dynamic KCl-stimulated insulin secretion (KSIS) analysis were scraped off from plates and relocated on to 24-mm insert and 3.0-μm polycarbonate membrane, six-well tissue culture, trans-well plate into hemispherical colonies, and the cells for insulin content analysis and flow cytometry analysis were kept on the original plates. All the cells were then further matured in MCDB131 medium supplemented with 1.5 g l^−1^ of sodium bicarbonate, 1× GlutaMAX, 20 mM glucose, 2% BSA, 100 nM LDN193189, 1 μM triiodothyronine (T3), 10 μM zinc sulfate, 10 μg ml^−1^ of heparin, 100 nM GS and 0.5× ITS-X for 7 d. Then cells were further matured in MCDB131 medium supplemented with 1.5 g l^−1^ of sodium bicarbonate, 1× GlutaMAX, 20 mM glucose, 2% BSA, 1 μM T_3_, 10 μM zinc sulfate, 10 μg ml^−1^ of heparin, 1 mM acetylcysteine, 10 μM Trolox, 2 μM R428 and 0.5× ITS-X with GENtoniK or control treatment for 7 d.

### Small-molecule treatment

A bioactive compound library containing 2,688 compounds was used for screening at a concentration of 5 μM (Selleck Bioactive Library, Selleck Chemicals). For confirmation of primary hits, compounds were extracted from the library plates with a JANUS (Perkin Elmer) liquid-handling platform and re-subjected to the high-content assay in triplicates at 5 μM. The 22 confirmed compounds were purchased from Selleck Chemicals, reconstituted in a suitable solvent and applied for dose–response validation in a concentration log scale (30 nM, 100 nM, 300 nM, 1000 nM, 3000 nM, 10,000 nM). GENtoniK cocktail was defined as a mixture of four small molecules—GSK2879552, EPZ-5676, Bay K 8644 and NMDA—applied at a working concentration of 1 μM each. Stocks of individual GENtoniK ingredients were reconstituted in DMSO to 10 mM (GSK2879552, EPZ-5676 and Bay K 8644) or in water to 50 mM (NMDA) and stored at −20 °C until the day of the experiments.

### Immunostaining

#### Monolayer cultures

Cells were fixed in 4% paraformaldehyde in phosphate-buffered saline (PBS) for 30 m, permeabilized for 5 min in PBS with 0.1% Triton X-100, and blocked for 30 min in PBS with 5% normal goat serum (NGS). Incubation with primary antibodies was performed overnight at 4 °C at the specified dilution in PBS with 2% NGS. After three washes with PBS, cells were incubated with fluorescently conjugated secondary antibodies (2 µg ml^−1^) and DAPI (1 µg µl^−1^) for 30 min at room temperature. For high-content experiments, all steps were assisted by automated liquid handling at the MSKCC Gene Editing and Screening Core Facility. A list of antibodies used in the present study is presented in Supplementary Table 3.

#### Forebrain organoids

Organoids were collected in 1.5-ml centrifuge tubes, washed in PBS and fixed with 4% paraformaldehyde solution in PBS overnight at 4 °C. Fixed organoids were rinsed in PBS and equilibrated in a solution of 30% w:v sucrose in PBS for 24 h or until they sank to the bottom of the tube. Organoids were embedded in optimal cutting temperature compound (Thermo Fisher Scientific) on cryomolds, flash frozen and sectioned to a thickness of 30 μm on a cryostat. Sections were collected in 1-ml centrifuge tubes, washed in Tris-buffered saline (TBS) with 0.3% Triton X-100 and blocked in the same solution with 10% NGS. Primary antibody incubation was done overnight in TBS with 0.5% Tween-20, followed by washes and secondary antibody incubation for 2 h at room temperature in the same buffer. Sections were mounted on slides with ProLong medium (Thermo Fisher Scientific) and imaged on a Zeiss microscope equipped with a ×20 high numerical aperture objective and an Apotome optical sectioning system (Zeiss). For quantification of SYN1 puncta, images were batch analyzed using the Synapse Counter ImageJ plugin^72^.

### HCS

#### High-content maturity assay

Cortical neurons were seeded on PLO/Lam-coated, 384-well plates at a density of 5,000 per well and maintained as described. For bioactive compound screening, compounds were added in fresh medium 7 d after plating to a final concentration of 5 μM in replicate plates. After 7 d of treatment, cells were rinsed twice and maintained in plain medium for an additional 7 d. Before fixation, one replicate plate was stimulated with 50 mM KCl for 2 h. Immunostaining for FOS, EGR-1 and MAP2 and counterstaining with DAPI were performed as described above. Images (four fields per well at ×20 magnification) were captured through an InCell Analyzer 6000 HCA system (GE Healthcare).

#### Image analysis and quantification of screen results

Phenotypic analysis of screen images was conducted in automated and blinded fashion using the Columbus software (Perkin Elmer). Extracted parameters included total number of nuclei, nuclear area, nuclear roundness index (DAPI), total neurite length per nucleus (MAP2) and fraction of FOS^+^, EGR-1^+^ and double-IEG-positive nuclei (FOS^+^/EGR-1^+^). For IEG quantification, ratios of positive nuclei were calculated by applying a threshold to arbitrary fluorescence units (AFU) within DAPI-positive nuclei (that is, >800 AFU for FOS, >1,000 AFU for EGR-1). IEG nuclei ratios in unstimulated plates were then subtracted from KCl-stimulated plates to isolate the KCl depolarization-mediated response. Morphological variables (nuclear and neurite) were averaged between unstimulated and KCl plates. Sequential *b*-score and *z*-score normalization and PCA were performed in the KNIME analytics platform^73^ with the High Content Screening Tools extension.

#### Synaptic marker analysis

Human PSC cortical neurons were plated on PLO/Lam 96-well plates. Drug treatment was initiated after 7 d and maintained for 21 d. Cells were fixed after an additional 7 d in plain medium. Immunostaining for SYN1, PSD95 and MAP2 was conducted as described above. Ten images per well were captured using the confocal modality of the InCell 6000 HCA system. A mask was applied to the area surrounding MAP2^+^ processes, and SYN1 and PSD95 puncta were quantified within the defined region. For quantification of pre- and postsynaptic marker apposition, a mask was applied to an area containing and immediately surrounding SYN1 puncta and PSD95 puncta localized within this region were counted. Synaptic puncta counts per field were normalized to total neurite length.

### Cell viability assays

#### Cytotox Red assay

Live imaging for quantification of live/dead cells was conducted using an Incucyte S3 platform (Sartorius) in cortical neurons plated on 96-well plates. Small-molecule treatment and loading with 250 nM Cytotox Red dye were initiated on day 7 after plating and replenished every 7 d. Images were captured every 2 h for 21 d. Analysis was performed using the Incucyte software.

#### Presto Blue assay

Presto Blue viability reagent (Thermo Fisher Scientific) was diluted in Neurobasal medium at 1:10. Viability experiments were performed in 96-well, black wall/clear bottomed plates. Then, 85 μl of Presto Blue/NB solution per well was incubated at 37 °C 5% CO_2_ for 2 h, after which 75 μl was transferred to a new plate for absorbance readings at 570 nm and 600 nm. Results were calculated by subtracting the 600-nm absorbance values from the 570-nm values, followed by subtracting background (600 − 570 nm) readings (read from blank wells) from experimental samples. GENtoniK and DMSO-treated neurons were compared at 1, 7 and 14 d of treatment to determine relative viability.

### Western blotting

Cells were harvested in radioimmunoprecipitation assay (RIPA) buffer and protein content quantified with Precision Red Assay. Protein, 10 μg, was loaded per lane, separated using a sodium dodecylsulfate–polyacrylamide gel electrophoresis system, and transferred on to a poly(vinylidene fluoride) membrane. A Gel Doc imaging system was used to visualize the blots. For visualizing 4R tau, a SuperSignal West Femto Maximum Sensitivity ECL Substrate (Thermo Fisher Scientific) was used. Quantification of band densities was performed in Image Lab and ImageJ.

### Shallow WGS and CNA analysis

Genomic DNA was collected using the Quick-DNA Miniprep Kit (Zymo) and submitted to the MSKCC Integrated Genomics Operation core for library preparation and shallow WGS. Paired-end reads were aligned to the GRCh19 reference human genome using BowTie2. Somatic CNA analysis was performed in R using the CNAclinic package (https://github.com/sdchandra/CNAclinic).

### Electrophysiology

#### Whole-cell patch-clamp

Human PSC cortical neurons were plated on to PLO/Lam-coated 35-mm dishes at a density of 75,000 cm^−2^. Treatment with GENtoniK or DMSO began 7 d after plating and maintained for 14 d. Recordings were initiated 7 d after treatment withdrawal, within 28–33 d of plating. Whole-cell recordings were performed at 23–24 °C while the cells were perfused in freshly made artificial cerebrospinal fluid (ACSF) containing (in mM): 125 NaCl, 2.5 KCl, 1.2 NaH_2_PO_4_, 1 MgSO_4_, 2 CaCl_2_, 25 NaHCO_3_ and 10 d-glucose. Solutions were pH corrected to 7.4 and 300–310 mOsmol. Neurons were recorded with pipettes of 3- to 7-MΩ resistance filled with a solution containing (in mM): 130 potassium gluconate, 4 KCl, 0.3 (ethylenebis(oxonitrilo))tetra-acetate (EGTA), 10 Na_2_ phosphocreatine, 10 Hepes, 4 Mg_2_ ATP, 0.3 Na_2_ GTP and 13 biocytin, pH adjusted to 7.3 with KOH and osmolarity to 285–290 mOsmol kg^−1^. Recordings were performed on a computer-controlled amplifier (MultiClamp 700B Axon Instruments) and acquired with an AxoScope 1550B (Axon Instruments) at a sampling rate of 10 kHz and low-pass filtered at 1 kHz. Recordings for sEPSCs were performed at 28–30 °C in a standard oxygenated ACSF. Neurons were recorded with pipettes of 3- to 7-MΩ resistance filled with a solution containing (in mM): 120 CsMeSO_4_, 8 NaCl, 0.3 EGTA, 10 tetraethylammonium Cl, 10 Hepes, 2 Mg_2_ ATP, 0.3 Na_2_ GTP, 13 biocytin and 3 QX-314-Cl, pH adjusted to 7.3 with KOH and osmolarity to 285–290 mOsmol kg^−1^. Membrane potentials were held at −60 mV or 0 mV to isolate AMPAR- or NMDAR-mediated events, respectively.

#### Multielectrode array recording

The hPSC-derived SMNs were seeded on to poly(l-lysine)-coated, complementary, metal oxide semiconductor, multielectrode array (CMOS-MEA) probes (3Brain)^74^. A 100-μl droplet of medium containing 200,000 neurons was placed on the recording area. After a 1-h incubation, 1.5 ml of medium was added to the probe and replaced every 3 d. Cells received treatment with GENtoniK or DMSO over days 3–9 after plating. Recordings were performed every 3 d for 18 d, 24 h after medium changes. Spontaneous activity (1 min) was sampled from 4,096 electrodes using the BioCAM system and analyzed using BrainWave 4 software. Spikes were detected using a precise timing spike detection algorithm^75^ on the raw channel traces, applying a threshold for detection of 9 s.d. Network bursts were detected by applying a hard threshold of 1 spike s^−1^ on the entire 4,096-channel array.

### Calcium imaging

Forebrain organoids were collected in 0.6-ml centrifuge tubes and rinsed 3× with Hepes-buffered Hanks’ balanced salt solution. Organoids were incubated in dye-loading solution consisting of 5 μM Fluo-4 AM (Thermo Fisher Scientific) with 0.1% Pluronic F-127 (Sigma-Aldrich), at 37 °C on an orbital shaker for 1 h. After incubation, organoids were rinsed in imaging solution consisting of modified Tyrode medium, as described previously^76^. Organoids were individually mounted on microscope slides fitted with customized adapters and sealed with no. 1.5 glass coverslips. Imaging was performed using a Nikon A1R HD25 confocal laser-scanning microscope equipped with a ×20 multiple immersion objective and a Tokai Hit stagetop incubator for temperature and CO_2_ control. Frames were captured every 5 s for 20 min (240 frames). Data analysis was performed using the CALIMA open-source software^77^.

### Gene expression and chromatin profiling

#### RNA-seq

RNA was extracted using the Direct-zol RNA miniprep kit (Zymo). Total RNA samples were submitted to GENEWIZ for paired-end sequencing at 30–40 million reads. Analysis was conducted on the Galaxy platform^78^. Transcript quantification was performed directly from adapter-trimmed FASTQ files using the Salmon quasi-mapping tool^79^ referenced to GENCODE Release 36 (GRCh38.p13) transcripts. DESeq2 (ref. ^80^) was used for differential expression analysis from Salmon-generated transcript per million (TPM) values. Differentially expressed genes with a Benjamini–Hochberg-adjusted *P* value <0.05 and a baseMean cutoff of 1,000 were applied to gene-set overrepresentation analysis using the Goseq tool^81^. For gene-set enrichment analysis (GSEA), all genes with a baseMean >1,000 were analyzed using the GSEA software^82^.

#### CUT&RUN

The hPSC-derived cortical neurons were collected 7 d after plating for CUT&RUN (cleavage under targets & release using nuclease) chromatin profiling using the standard protocol^83^. Antibodies against H3K4me2 (Upstate), H3K79me2 (Active Motif) and mouse immunoglobulin (Ig)G (Abcam) were used at 1:100 for 100,000 cells per antibody. DNA was collected via phenol–chloroform extraction and submitted to the MSKCC Integrated Genomics Operation core for paired-end sequencing at 5 million reads. Analysis was performed in the Galaxy platform. After alignment to ENSEMBL GRCh38 genome build using Bowtie2 (ref. ^84^), peaks were called using MACS^85^ and visualized with ChIPSeeker^86^ and deepTool2 (ref. ^87^), with mouse IgG as a control for normalization.

### Flow cytometry

The hPSC neurons were dissociated to single-cell suspensions using Accutase (Innovative Cell Technologies) supplemented with Neuron Isolation Enzyme for Pierce (Thermo Fisher Scientific) solution at 1:50. Single-cell suspensions were stained with Zombie UV Fixable Viability Kit (BioLegend) at 1:2,500 in PBS for 15 min (room temperature), followed by fixation in 4% paraformaldehyde for 10 min (4 °C), then permeabilized in 0.5% Triton X-100 for 10 min (4 °C) and blocked in 5% BSA for 10 min (4 °C). Cells were stained with H3K9me3-PE antibody (Cell Signaling Technologies) diluted 1:200 for 30 min at 4 °C and acquisition was performed on the Cytek Aurora Spectral Flow Cytometer, with data analyzed on FlowJo v.10.8.1.

### Dot blot for melanocyte pigmentation

The hESC melanocytes were dissociated in Accutase, rinsed and collected in PBS. A pellet containing 1 M cells was lysed in 50 μl of RIPA buffer with sonication and centrifuged at 10,000*g* for 3 min. After discarding the supernatant, the insoluble fraction was resuspended in 80 μl of PBS. Then, 10 μl of this solution was applied to a nitrocellulose membrane, air dried and imaged with a standard office scanner to assess pigmentation.

### Pancreatic β-cell maturation assays

#### Flow cytometry analysis

The hESC-derived cells were dissociated using Accutase, fixed and permeabilized using Fixation/Permeabilization Solution Kit (BD Biosciences) according to the manufacturer’s instructions. Briefly, cells were first fixed with fixation/permeabilization buffer for 30 min at 4 °C in the dark and then washed twice with washing buffer with a 10-min incubation each time at room temperature. Then, the fixed cells were incubated with primary antibody overnight at 4 °C and washed twice with washing buffer with a 10-min incubation each time at room temperature. After a 30-min incubation with fluorescence-conjugated secondary antibody at 4 °C, cells were washed twice with washing buffer with a 10-min incubation each time at room temperature and resuspended in PBS buffer for analysis. The following primary antibodies were used: anti-insulin (1:50, Dako) and anti-glucagon (1:100, Abcam). Samples were analyzed with an Accuri C6 flow cytometry instrument and the data were processed using FlowJo v.10 software.

#### Static and dynamic KSIS

On day 30, cells were starved in 2 ml of glucose-free, pancreatic β-cell maturation medium and followed by 2 ml of glucose-free DMEM (with GlutaMAX) for 1 h and an additional 1-h incubation in KRBH buffer (containing 140 mM NaCl, 3.6 mM KCl, 0.5 mM NaH_2_PO_4_, 0.2 mM MgSO_4_, 1.5 mM CaCl_2_, 10 mM Hepes, pH 7.4, 2 mM NaHCO_3_ and 0.1% BSA) in a 5% CO_2_/37 °C incubator. To perform static KSIS, cells were exposed sequentially to 100 μl of KRBH with 2 mM glucose or 2 mM glucose with 30 mM KCl; supernatants were collected after 60 min and spun down to eliminate the cells and debris. Supernatants were used for ELISA (Insulin Chemiluminescence ELISA Jumbo). To measure the total insulin levels in cells in each sample, cells were lysed in RIPA buffer supplemented with 1× Protease Inhibitor Cocktail (Thermo Fisher Scientific) with vortexing for 2 min at room temperature and flash freezing the samples in liquid nitrogen and thawing to help lysis and release the cellular insulin. Lysates were spun down and the supernatant was used for ELISA. Insulin secretion from cells in each condition was normalized to KRBH treatment. To perform dynamic KSIS, cells were embedded in chambers with the order of a filter paper–biogel P4 beads–cells–biogel P4 beads sandwich and then the chambers were installed on the biorep perfusion system (Biorep Technology) and first perfused with Krebs’ buffer containing 2 mM glucose at a flow rate of 100 μl min^−1^ and followed by perfusion with 2 mM glucose + 30 mM KCl for 25 min. Insulin secretion from cells in each fraction in KCl stimulation was normalized to KRBH treatment.

#### Insulin content measurement

Day-30 hESC-derived β-like cells were dissociated using Accutase and resuspended in DMEM containing 2% FBS and 1 mM EDTA. *INS*-GFP^+^DAPI^−^ cells, 80,000, were FACS sorted by an ARIA2 instrument, washed once with PBS and lysed in 200 µl of RIPA buffer supplemented with 1× Protease Inhibitor Cocktail (Thermo Fisher Scientific). The insulin content was measured by ELISA.

#### Immunoelectron microscopy

To analyze granular ultrastructure, control or chemically treated, hPSC-derived, β-like cell clusters were washed with serum-free medium and fixed with 2.5% glutaraldehyde, 4% paraformaldehyde and 0.02 % picric acid in 0.1 M buffer. After three buffer washes, the cell clusters were fixed again using 1% OsO_4_–1.5% potassium ferricyanide at room temperature for 60 min, followed by three buffer washes. After dehydration steps of 50%, 70%, 85%, 95%, 100%, 100% and 100% EtOH, the cell clusters were infiltrated with 100% EtOH mixed 1:1 with acetonitrile, followed by acetonitrile, acetonitrile 1:1 with Embed 812 epoxy resin, resin and, finally, embedded in fresh resin which was polymerized at 50 °C for 36 h. Sections were cut at 65 nm and picked up on nickel grids. Sections were washed with saturated sodium periodate, followed by 50 mM glycine and blocking buffer. Then, the sections were stained with anti-insulin antibody at the original dilution followed by 10-nm gold goat anti-guinea pig IgG (Aurion, 1:100). Samples were imaged with a JEOL JEM 1400 transmission ekectron microscope with an Olympus-SIS 2,000 × 2,000 Veleta CCD camera.

### Statistical analysis

Averages are reported as arithmetic means ± s.e.m. unless otherwise indicated. Statistical significance was marked by asterisk notation as follows: NS: *P* > 0.05, *P ≤ 0.05, **P ≤ 0.01, ***P ≤ 0.001, ****P ≤ 0.0001. Biological replicates are defined as independent differentiations of a given hPSC line unless indicated otherwise. Statistical tests were performed using Graphpad Prism 9.1.

### Reporting summary

Further information on research design is available in the Nature Portfolio Reporting Summary linked to this article.

## Online content

Any methods, additional references, Nature Portfolio reporting summaries, source data, extended data, supplementary information, acknowledgements, peer review information; details of author contributions and competing interests; and statements of data and code availability are available at 10.1038/s41587-023-02031-z.

## Supplementary information

Supplementary Information Supplementary Figs. 1–20.

Reporting Summary

Supplementary Tables Supplementary Table 1 Hit list. Supplementary Table 2 Experimental details. Supplementary Table 3 Antibodies.

Supplementary Video 1 Cortical organoid treated with DMSO, stained with calcium Fluo-4 AM, to image spontaneous calcium spikes.

Supplementary Video 2 Cortical organoid treated with GENtoniK, stained with calcium Fluo-4 AM, to image spontaneous calcium spikes.

Supplementary Video 3 Multielectrode array recording of SMNs treated with GENtoniK.

Supplementary Data 1 Statistical source data for supplementary figures.

## Source data

Source Data Fig. 1 Statistical source data.

Source Data Fig. 2 Statistical source data.

Source Data Fig. 3 Statistical source data.

Source Data Fig. 4 Statistical source data.

## Data Availability

Data generated during the present study are deposited at the National Center for Biotechnology Information Gene Expression Omnibus under accession nos. GSE172544 (RNA-seq) and GSE172543 (CUT&RUN). GRCh37 hg19 is publicly available at https://www.ncbi.nlm.nih.gov/datasets/genome/GCF_000001405.13 and GRCh37 p13 at https://www.ncbi.nlm.nih.gov/datasets/genome/GCF_000001405.39. Brainspan reference data are available at Brainspan.org. Source data are provided with this paper.
