# Author Correction: Combined small-molecule treatment accelerates maturation of human pluripotent stem cell-derived neurons

**DOI:** 10.1038/s41587-024-02233-z

**Published:** 2024-05-21

**Authors:** Emiliano Hergenreder, Andrew P. Minotti, Yana Zorina, Polina Oberst, Zeping Zhao, Hermany Munguba, Elizabeth L. Calder, Arianna Baggiolini, Ryan M. Walsh, Conor Liston, Joshua Levitz, Ralph Garippa, Shuibing Chen, Gabriele Ciceri, Lorenz Studer

**Affiliations:** 1 The Center for Stem Cell Biology, Sloan–Kettering Institute for Cancer Research, New York, NY USA; 2 Developmental Biology Program, Sloan–Kettering Institute for Cancer Research, New York, NY USA; 3 Weill Graduate School of Medical Sciences of Cornell University, New York, NY USA; 4 Gene Editing and Screening Core Facility, Sloan–Kettering Institute for Cancer Research, New York, NY USA; 5 Department of Surgery, Weill Cornell Medicine, New York, NY USA; 6 Department of Biochemistry, Weill Cornell Medicine, New York, NY USA; 7 Department of Psychiatry, Weill Cornell Medicine, New York, USA

**Keywords:** Embryonic stem cells, Induced pluripotent stem cells, Differentiation, Cellular neuroscience, Neuronal development

Correction to: *Nature Biotechnology* 10.1038/s41587-023-02031-z, published online 2 January 2024.

In Fig. 3h–k we recorded spontaneous excitatory postsynaptic currents (sEPSCs) in human PSC-derived cortical neurons at either –60 mV or at 0 mV, comparing their synaptic properties following GENtoniK versus DMSO treatment. We reported those currents as either “AMPAR”- or “NMDAR”-mediated, respectively. However, those data are insufficient to make the claim of specifically measuring NMDAR currents without including more detailed analyses including pharmacological validation studies. Accordingly, we changed the text, Fig. 3h and the corresponding figure legend to reflect this limitation of our study. We thank the reader who brought this issue to our attention.

