# Supplementary material for: Combined small-molecule treatment accelerates maturation of human pluripotent stem cell-derived neurons
Source: Nat Biotechnol. 2024 Jan 2;42(10):1515–25. doi: 10.1038/s41587-023-02031-z (PMC11348887; doi:10.1038/s41587-023-02031-z)
Supplement: Supplementary file 1 — Supplementary Figs. 1–20. [file 41587_2023_2031_MOESM1_ESM.pdf]

nature biotechnology

Article https://doi.org/10.1038/s41587-023-02031-z

Combined small-molecule treatment accelerates maturation of human pluripotent stem cell-derived neurons

In the format provided by the authors and unedited

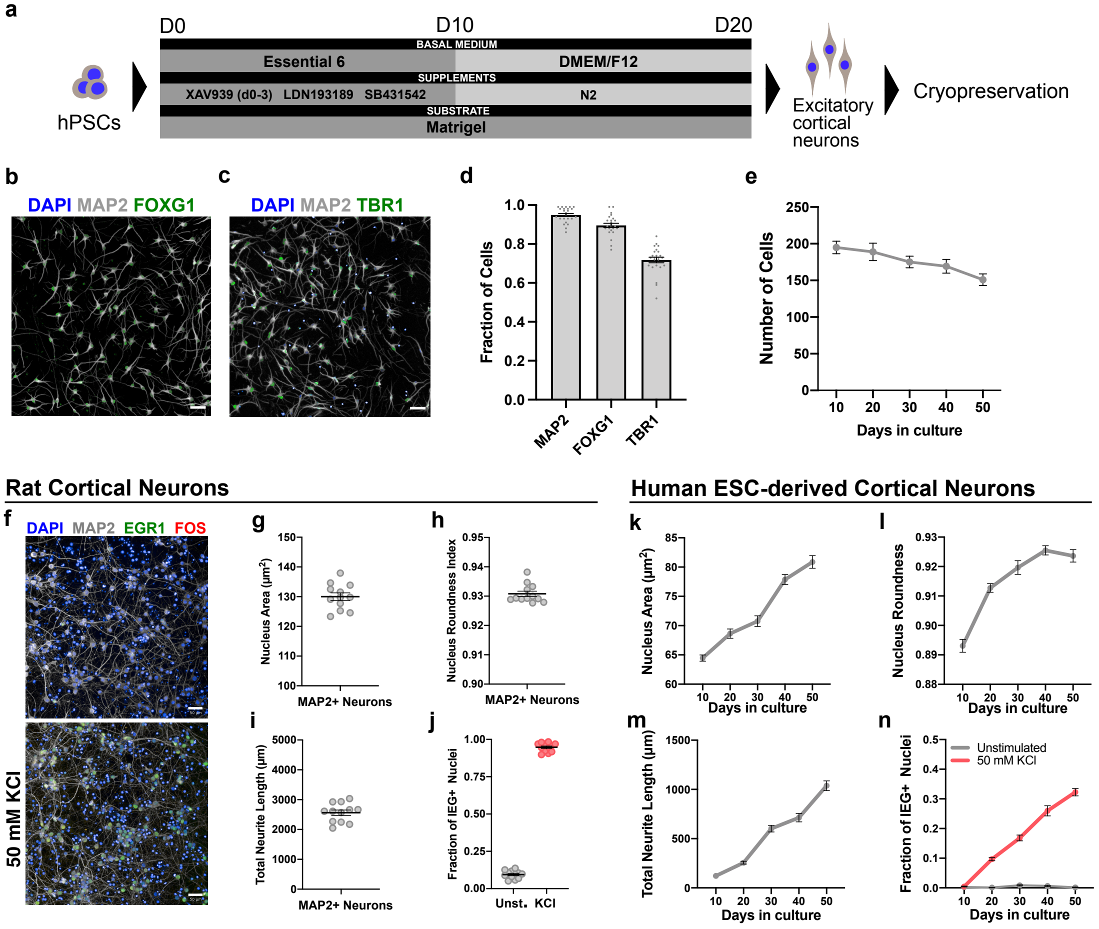

Supplementary Figure 1 - Design and optimization of high-content maturation assay. a*, Schematic showing protocol used to differentiate hPSCs into excitatory cortical neurons.* ***b-d****, immunofluorescent staining of day 10 hPSC-cortical neurons for pan-neuronal marker MAP2 (****b****,* ***c****), forebrain marker FOXG1 (****b****), and deep-layer cortex marker TBR1 (****c****).* ***d****, Quantification of immunofluorescent staining (n=12 microplate wells).* ***e,*** *Time-course quantification of cell number in post-mitotic hPSC-cortical neurons (DAPI+ cells per field, n=24 microplate wells).* ***f****, Immunofluorescent staining of primary embryonic rat cortex neurons (E18) using high-content markers.* ***g-j****, Quantification of maturation parameters in E18 primary rat neurons after 14 days in culture demonstrate mature values for nucleus size (****g****), nucleus roundness (****h****), neurite length (****i****), and KCl-induced IEG expression (****j****) (n = 12 microplate wells).* ***k-n****, Time course quantification of maturation parameters in hPSC-derived cortical neurons showing time-dependent increases in nucleus size (****k****), nucleus roundness (****l****), neurite length (****m****), and KCl-induced IEG expression (****n****) (n=24 microplate wells). Mean values are represented by a black line. Error bars represent S.E.M. Scale bars are 50 µm.*

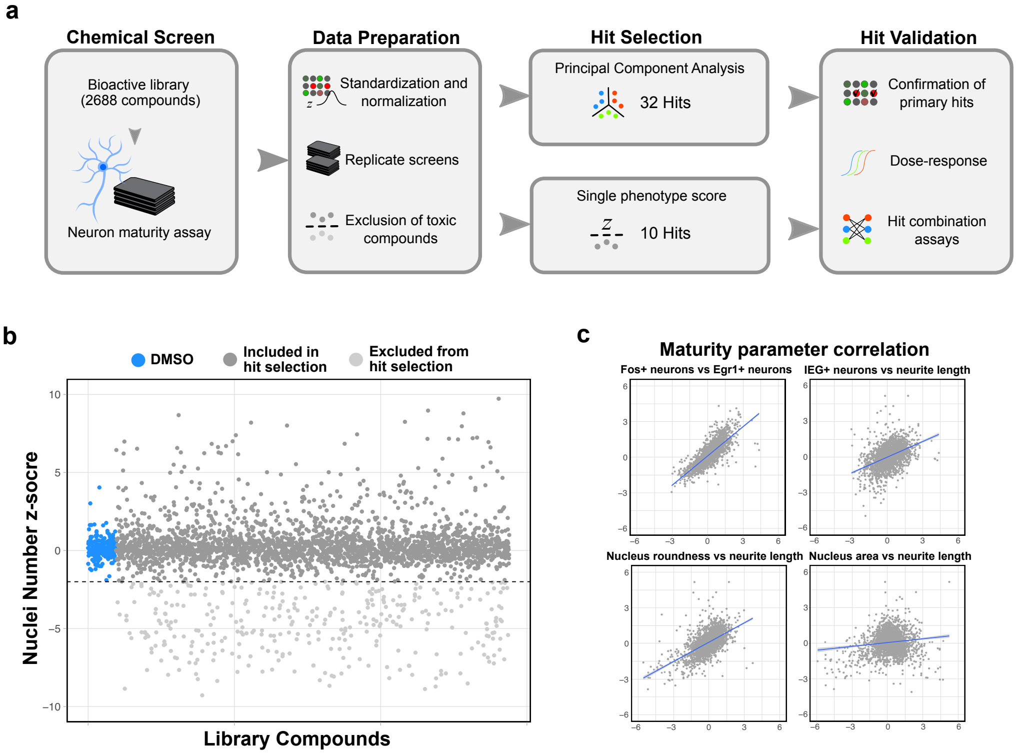

***Supplementary Figure 2 - High-content screen data preparation and analysis. a**, Pipeline of analysis of high-content screen using a 2688-compound bioactive library. Normalization scores (z-scores) of 2 independent screens were averaged and used for selection of hits via PCA or single-parameter scores. **b**, Exclusion of toxic compounds with a mean z-score of total cell number below -2. Note that increases in total cell number were only observed for compounds inducing non-neural cells (Fig. 1e). **c**, Correlation of mean maturation z-scores from 2 screen runs among non-toxic compounds.*

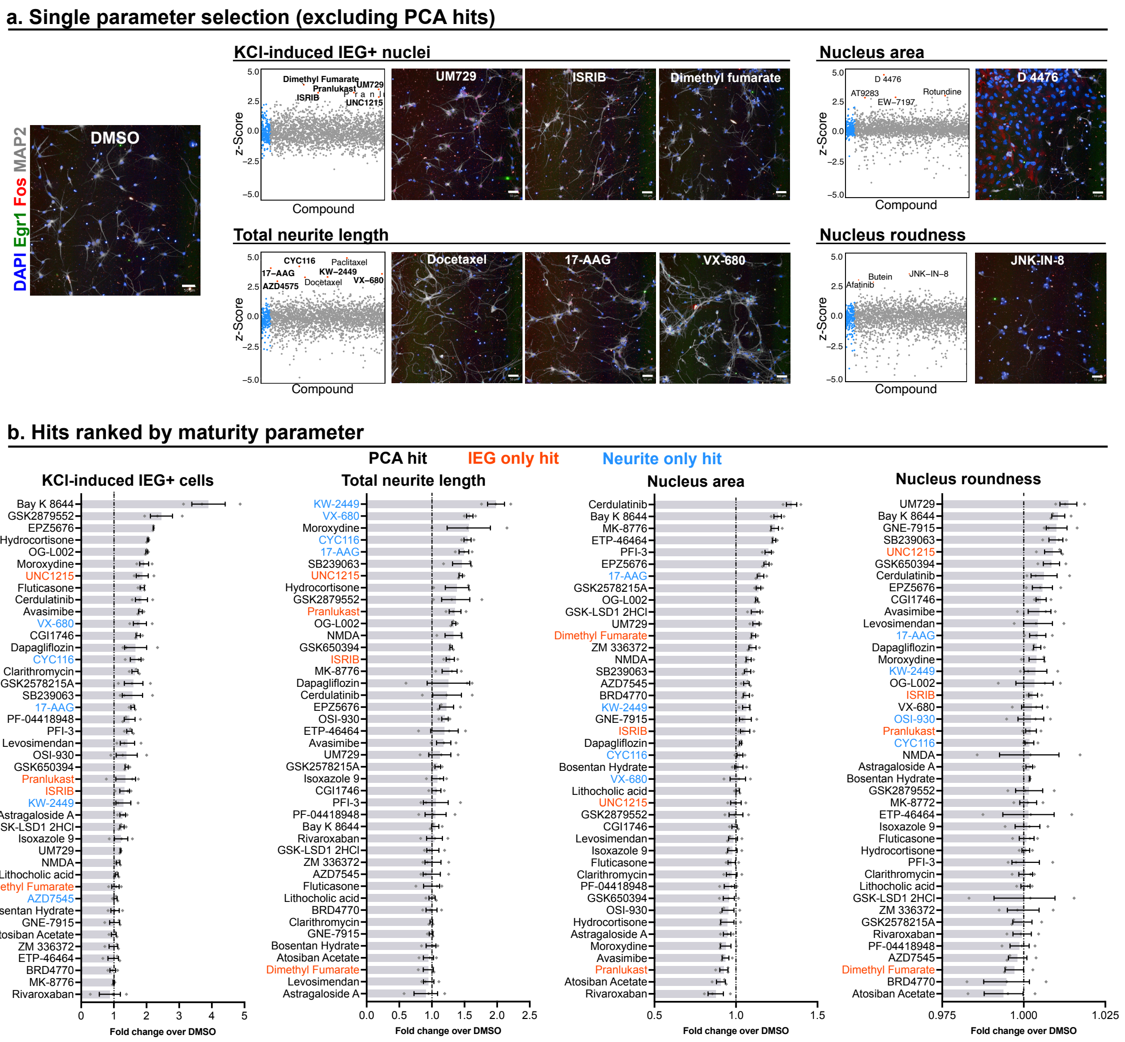

***Supplementary Figure 3 - Single parameter hit selection**. **a**, Left, representative high-content screen image of a DMSO control well. Right, library compounds (excluding the PCA hits already selected) plotted against individual maturation parameter. Selected compounds are highlighted in bold, non-highlighted compounds were not included due to phenotype and/or known molecular target unrelated to neuronal maturation. Screen images are representative of high-scoring compounds for each parameter. **b**, ranking of 42 primary hits (PCA and single parameter) in individual maturation parameters (n=3 microplate wells). Mean values are represented by bar graph. Error bars represent S.E.M. Scale bars are 50 µm.*

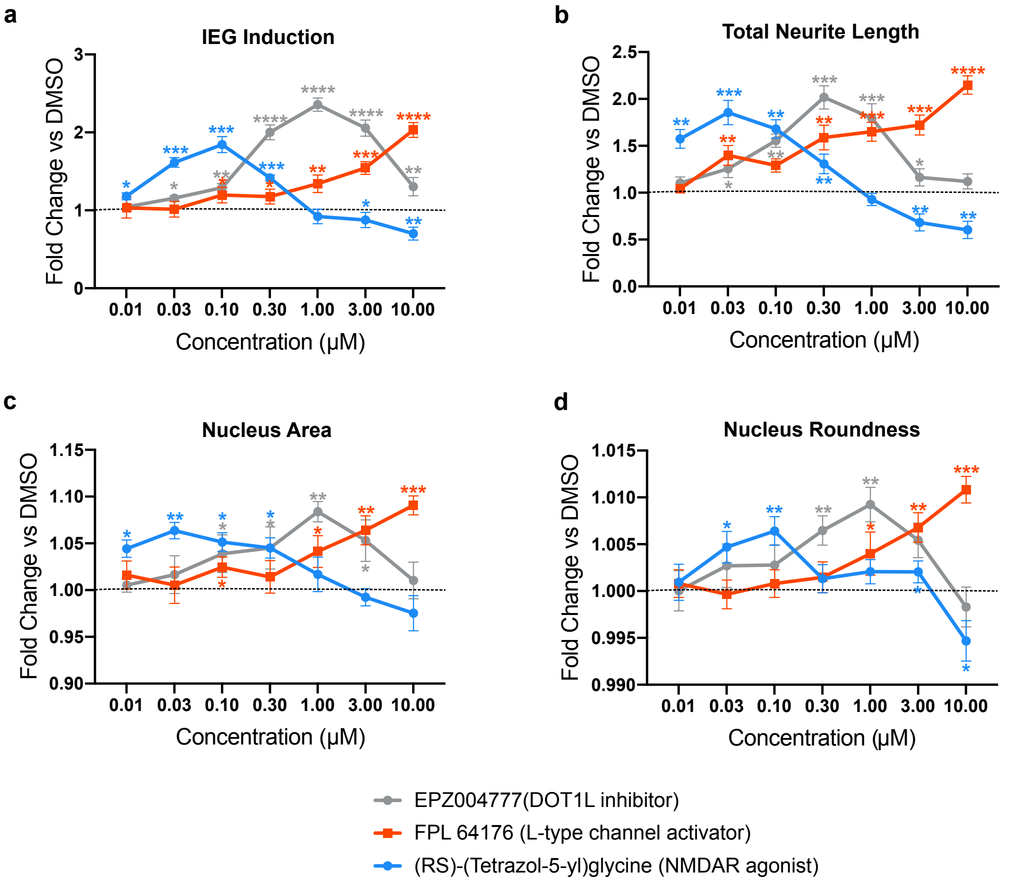

***Supplementary Figure 4 - Validation of screen hits with compounds with identical molecular targets**. **a-d**, Dose-response validation with an additional inhibitor of DOT1L (EPZ004777), l-type channel activator (FPL 64176), and NMDA receptor agonist ((RS)-(Tetrazol-5-yl) glycine) comparing IEG induction (**a**), total neurite length (**b**), nucleus area (**c**), and nucleus roundness (**d**), normalized to DMSO controls. Two-tailed Welch's t-tests were performed from unnormalized values and DMSO controls; asterisks indicate statistical significance. n = 32 microplate wells from 2 independent differentiations. Error bars represent S.E.M.*

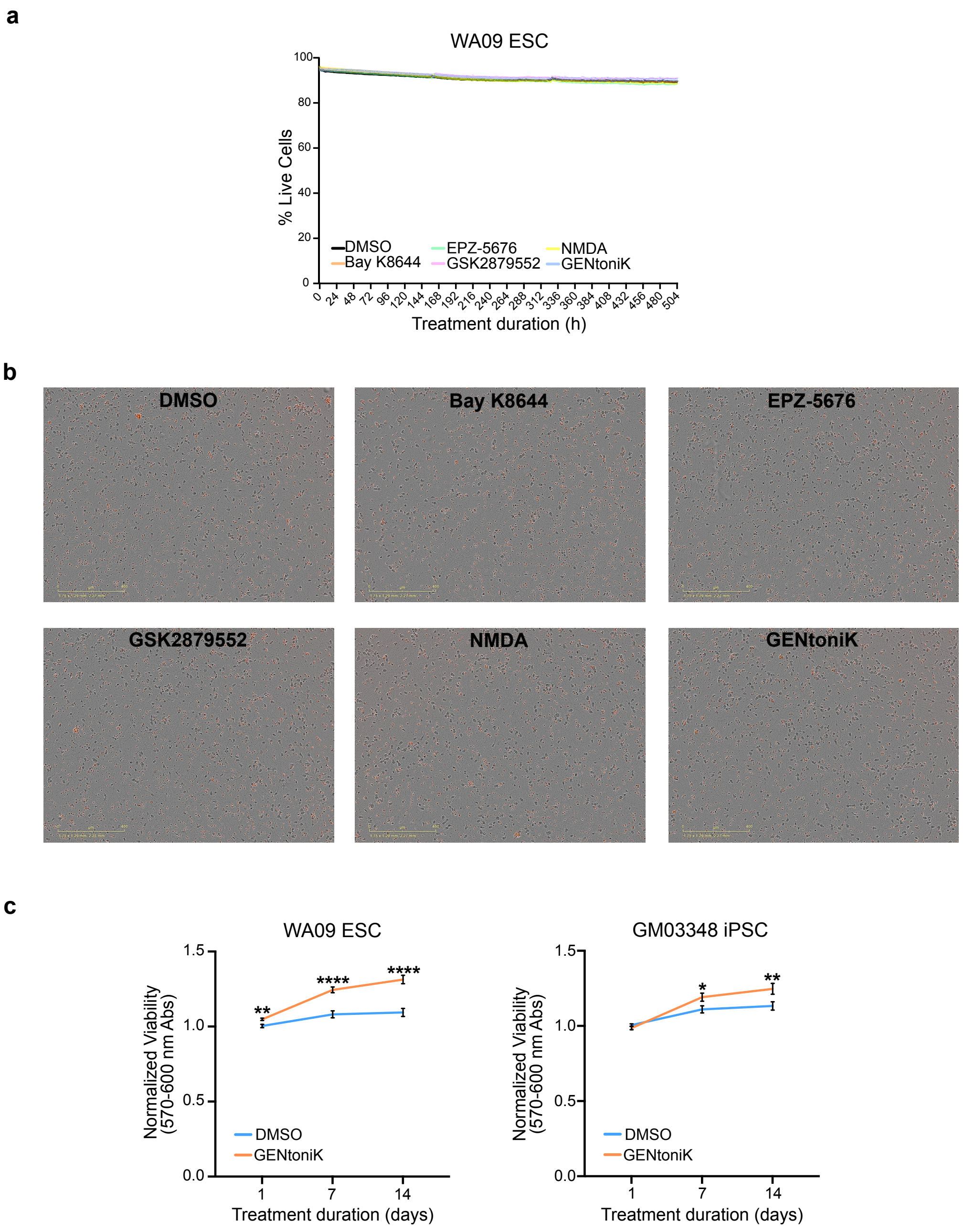

Supplementary Figure 5 - Chemical promoters of neuron maturation do not influence neuron viability. a, *Time-course analysis of plasma membrane integrity with Cytotox Red dye in neurons derived from hESC. Neurons were incubated in the presence of individual treatment or complete GENtoniK for 21 days (n=16 microplate wells).* ***b***, *Representative images of Cytotox Red-loaded neuron cultures on day 21 of treatment. Small Cytotox Red+ particles outside cells are debris that is present from plating and do not affect the quantification of live cells.* ***c***, *PrestoBlue assay of cell viability in neurons derived from two hPSC lines that received treatment with DMSO or GENtoniK for 1, 7, or 14 days (n=16 microplate wells), normalized to DMSO Day 1 570-600 nm absorbance. Two-tailed Welch's t-test; asterisks indicate statistical significance. Error bars represent S.E.M. Scale bars are 400 μm.*

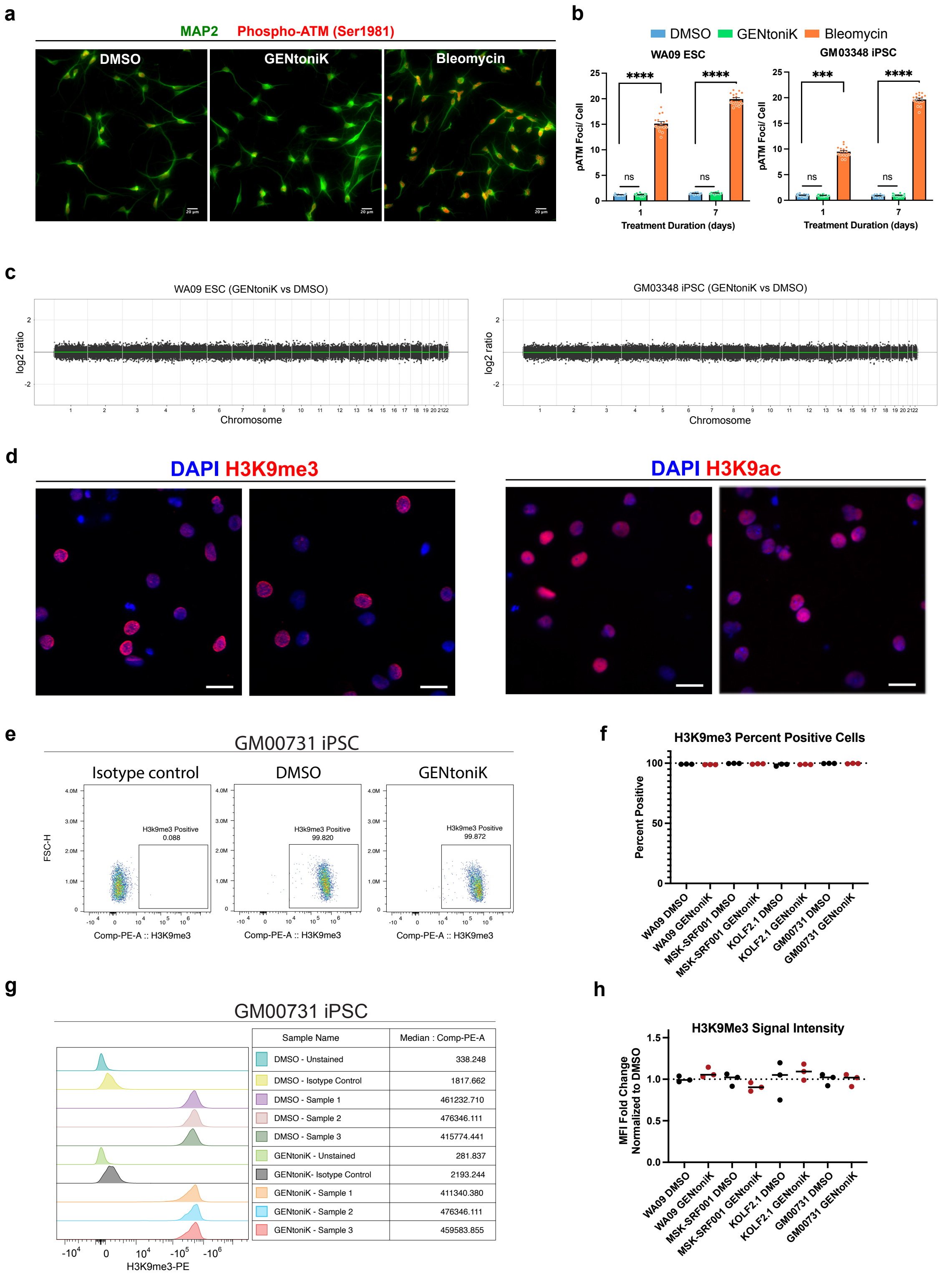
a
MAP2 Phospho-ATM (Ser1981)
DMSO
GENtoniK
Bleomycin
b
DMSO GENtoniK Bleomycin
WA09 ESC
GM03348 iPSC
pATM Foci/ Cell
Treatment Duration (days)
ns
****
***
c
WA09 ESC (GENtoniK vs DMSO)
GM03348 iPSC (GENtoniK vs DMSO)
log2 ratio
Chromosome
d
DAPI H3K9me3
DAPI H3K9ac
e
GM00731 iPSC
Isotype control
DMSO
GENtoniK
FSC-H
H3k9me3 Positive 0.088
H3k9me3 Positive 99.820
H3k9me3 Positive 99.872
Comp-PE-A :: H3K9me3
f
H3K9me3 Percent Positive Cells
Percent Positive
WA09 DMSO
WA09 GENtoniK
MSK-SRF001 DMSO
MSK-SRF001 GENtoniK
KOLF2.1 DMSO
KOLF2.1 GENtoniK
GM00731 DMSO
GM00731 GENtoniK
g
GM00731 iPSC
Sample Name | Median : Comp-PE-A
DMSO - Unstained | 338.248
DMSO - Isotype Control | 1817.662
DMSO - Sample 1 | 461232.710
DMSO - Sample 2 | 476346.111
DMSO - Sample 3 | 415774.441
GENtoniK - Unstained | 281.837
GENtoniK- Isotype Control | 2193.244
GENtoniK - Sample 1 | 411340.380
GENtoniK - Sample 2 | 476346.111
GENtoniK - Sample 3 | 459583.855
H3K9me3-PE
h
H3K9Me3 Signal Intensity
MFI Fold Change Normalized to DMSO
WA09 DMSO
WA09 GENtoniK
MSK-SRF001 DMSO
MSK-SRF001 GENtoniK
KOLF2.1 DMSO
KOLF2.1 GENtoniK
GM00731 DMSO
GM00731 GENtoniK

Supplementary Figure 6 - Chemical promoters of neuron maturation do not cause cell stress or induce DNA or chromatin damage. a*, Representative images of neurons stained for double strand break marker phospho-ATM after overnight treatment with DMSO, GENtoniK, or radiomimetic drug bleomycin.* ***b****, Quantification of phospho-ATM positive foci in neurons from 2 hPSC lines treated with DMSO, GENtoniK, or bleomycin for 1 or 7 days (n=16 microplate wells, data are arithmetic mean with error bars showing S.E.M., Two-Tailed Welch's t-test was used with asterisks indicating statistical significance).* ***c****, CNA analysis from shallow WGS results in neurons derived from two hPSC lines reveals no instances of copy number aberrations in GENtoniK-treated neurons compared to DMSO treated controls.* ***d****, Representative immunofluorescence staining for trimethylated (left) and acetylated (right) H3K9, showing no obvious changes in heterochromatin and active chromatin structure in GENtoniK-treated versus DMSO-treated cortical neurons after 14 days of treatment. Scale bars are 20 µm. (n=1 independent differentiation).* ***e,*** *Representative flow cytometry plots showing the H3K9me3 percent positive neurons, gated on an isotype control antibody, from the GM00731 iPSC line.* ***f,*** *Summary of H3k9me3-percent positive cells treated with either DMSO (black) or GENtoniK (red) across four hPSC lines. Each dot represents one technical replicate (n=3 technical replicates per n=4 biological replicates).* ***g,*** *Representative histograms showing H3K9me3 fluorescence intensity of DMSO or GENtoniK treated neurons with the Median Fluorescence Intensity (MFI) indicated in the right-hand table, of GM00731 iPSC neurons.* ***h,*** *Summary of H3K9me3 MFI in DMSO (black) or GENtoniK (red) treated neurons (n=3 technical replicates per 4 independent hPSC lines).*

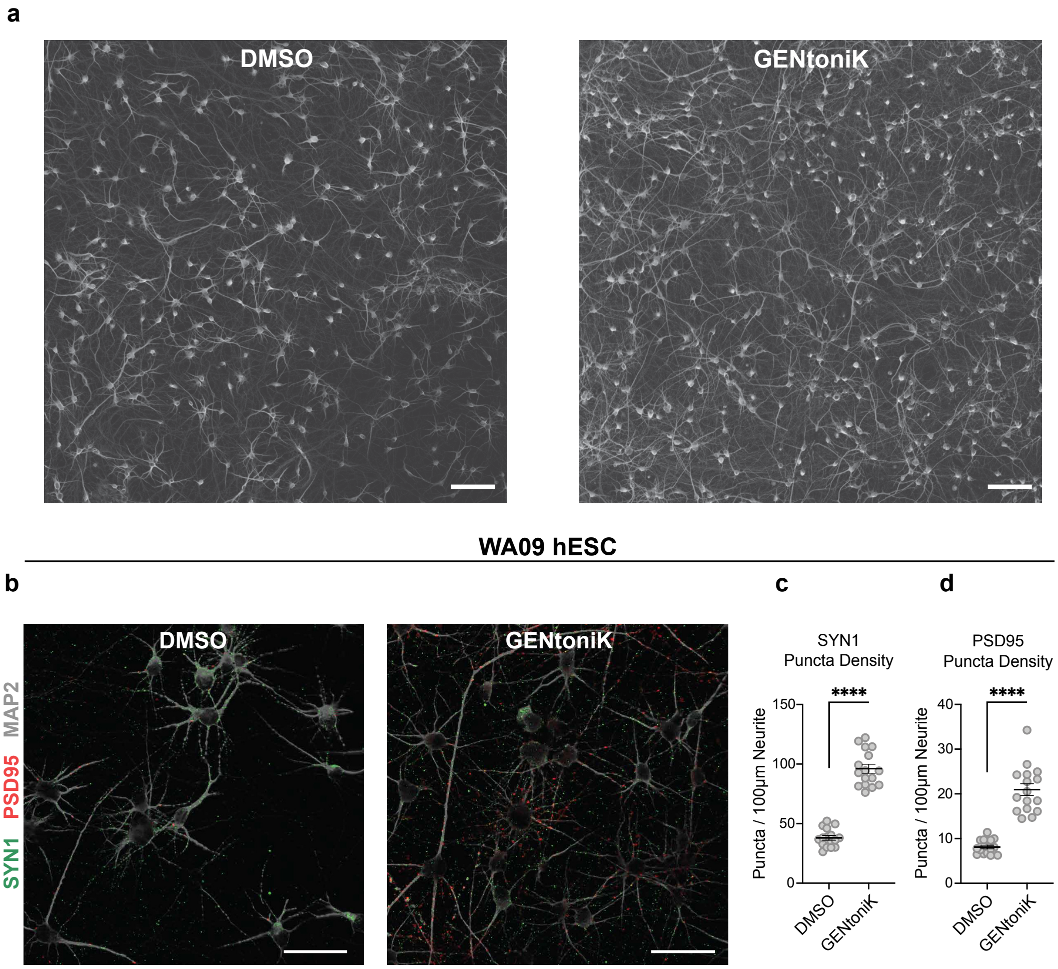

Supplementary Figure 7 - GENtoniK increases density of pre- and post- synaptic marker puncta. a*, Representative confocal microscopy images of immunostaining for dendritic marker MAP2 in day 35 hPSC-cortical neurons, showing increase in neurite growth and branching in GENtoniK-treated (right) vs DMSO-treated (left) neurons.* ***b****, Representative structured illumination (apotome) images of pre- and post-synaptic markers SYN1 and PSD95 in day 35 hPSC-cortical neurons.* ***b****-****c****, GENtoniK increases density of SYN1 (****b****) and PSD-95 (****c****) apposition expressed as puncta per neurite length (n=16 wells from n=2 independent differentiations). Two-tailed Welch's t-test; asterisks indicate statistical significance. Error bars represent S.E.M. Scale bars are 100 µm (****a****) and 20 µm (****b****).*

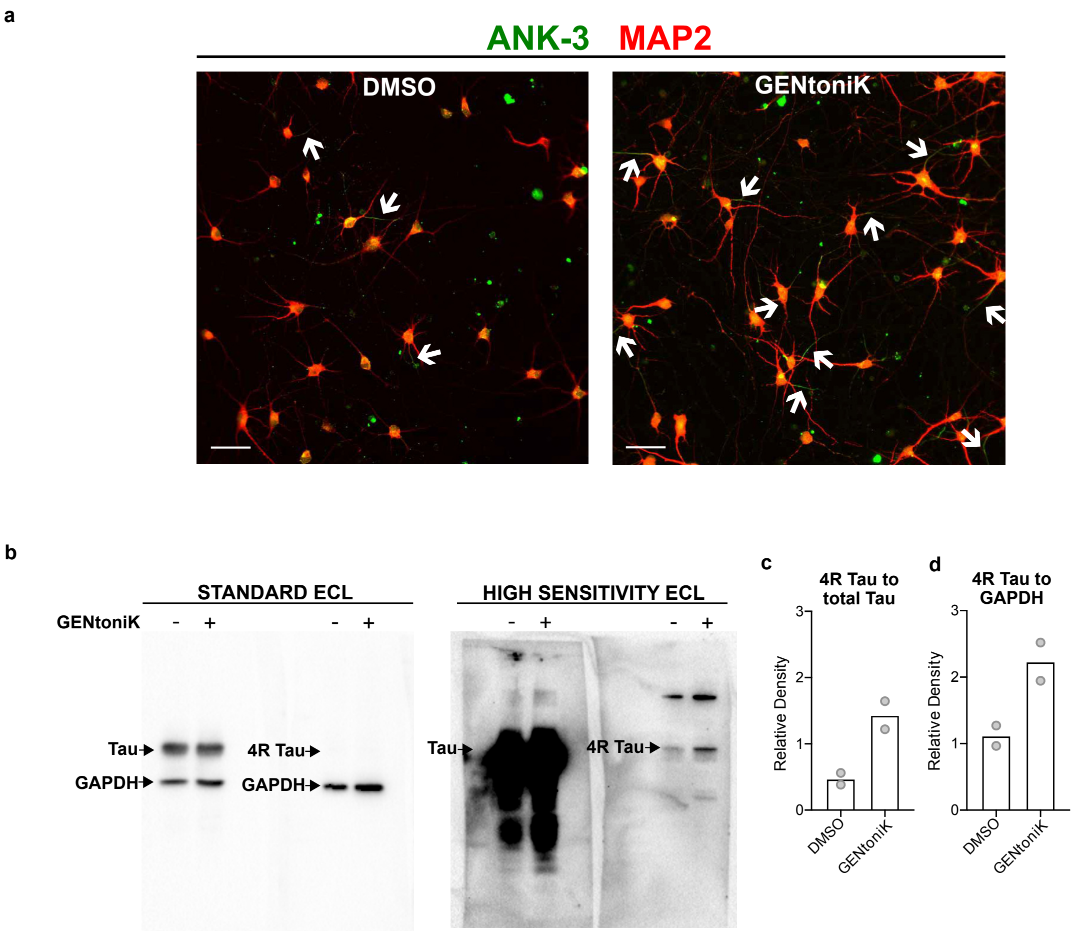

Supplementary Figure 8 - Effect of GENtoniK on axonal maturation. a*, Representative images of day 21 cortical neurons treated with DMSO and GENtoniK from days 7-14, stained for dendritic marker MAP2 and AIS marker ANK3. AIS are present even in immature, untreated neurons. Scale bars are 50 µm.* ***b****, representative western blots of day 28 neurons treated with DMSO or GENtoniK from days 7-21. Blots were probed for total Tau, 4-repeat Tau, and GAPDH, and visualized with standard (right) and high sensitivity (left) ECL reagent.* ***c*****-*****d****, band densitometry analysis revealing increased expression of 4-repeat tau in GENtoniK-treated neurons normalized to total Tau (****c****) and GAPDH (****d****) (n=2 independent differentiations).*

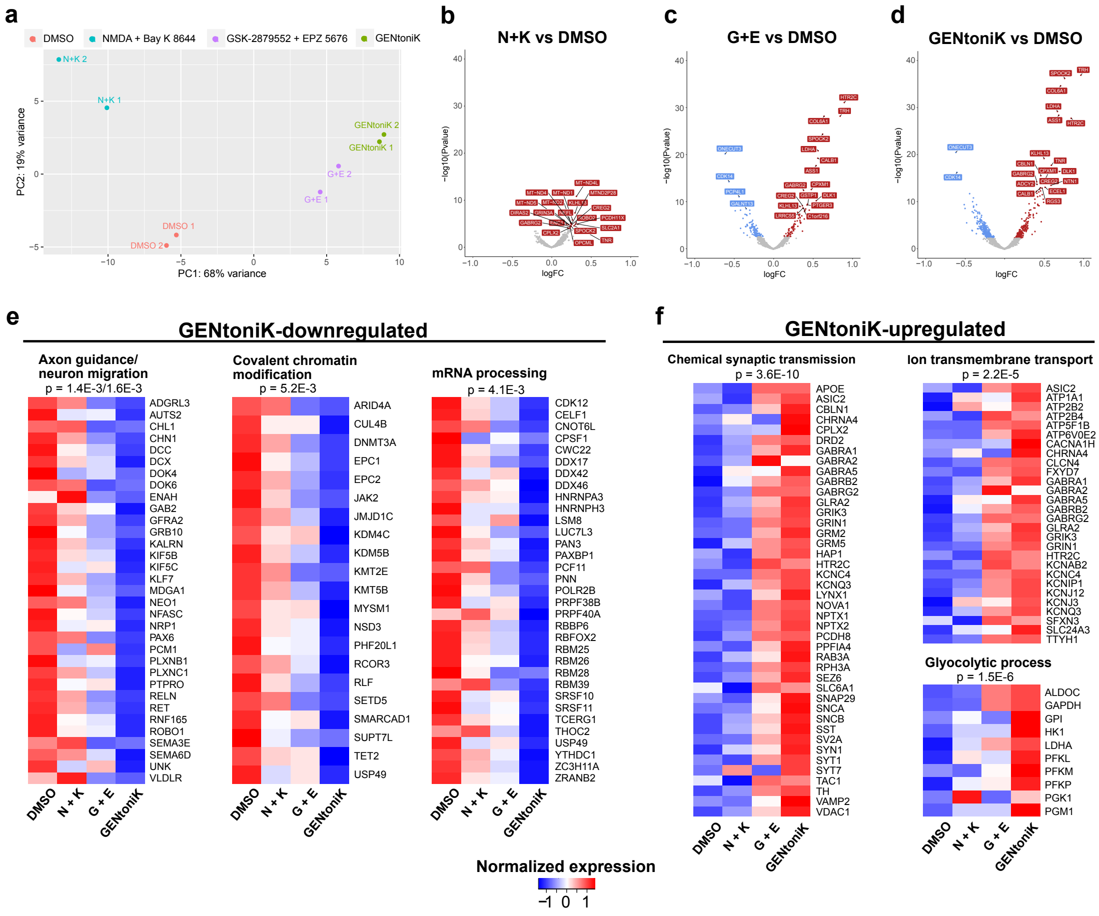

Supplementary Figure 9 - RNA-seq results of day 21 neurons treated with maturation promoting small molecules from d7-14. ***a****, Principal component analysis of RNA-seq results from neurons treated with DMSO, two epigenetic drugs (G+E), two calcium influx driving compounds (N+K), or complete GENtoniK.* ***b-d****, Volcano plots of RNA-seq differential expression analysis vs DMSO of calcium influx agonist NMDA and Bay K 8644 (****b****), epigenetic drugs GSK-2879552 and EPZ-5676 (****c****), or complete GENtoniK (****d****).* ***e, f,*** *Heatmaps of genes within overrepresented biological process ontology categories among GENtoniK-downregulated (****e****), and upregulated (****f****) genes. RNA-seq results from 3 independent differentiations. Heatmaps show expression normalized by row, calculated from mean TPM values. Displayed p-values are for enrichment of stated gene ontology categories among differentially expressed transcripts using the Wald Test.*

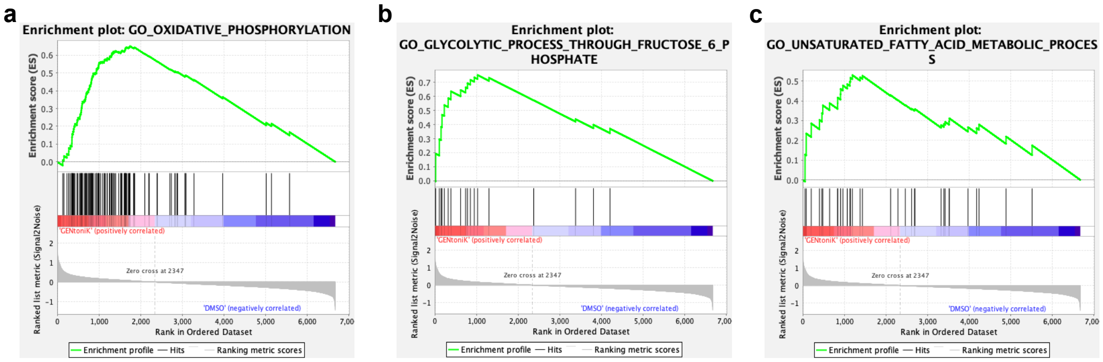

Supplementary Figure 10 - GENtoniK induces transcriptional activation of diverse metabolic pathways in cortical neurons. a-c, *Gene set enrichment analysis (GSEA) of RNA-seq results showing enrichment for oxidative phosphorylation (**a**), canonical glycolysis (**b**), and fatty acid metabolism (**c**) gene ontology categories enriched in GENtoniK-treated neurons. n=3 independent differentiations.*

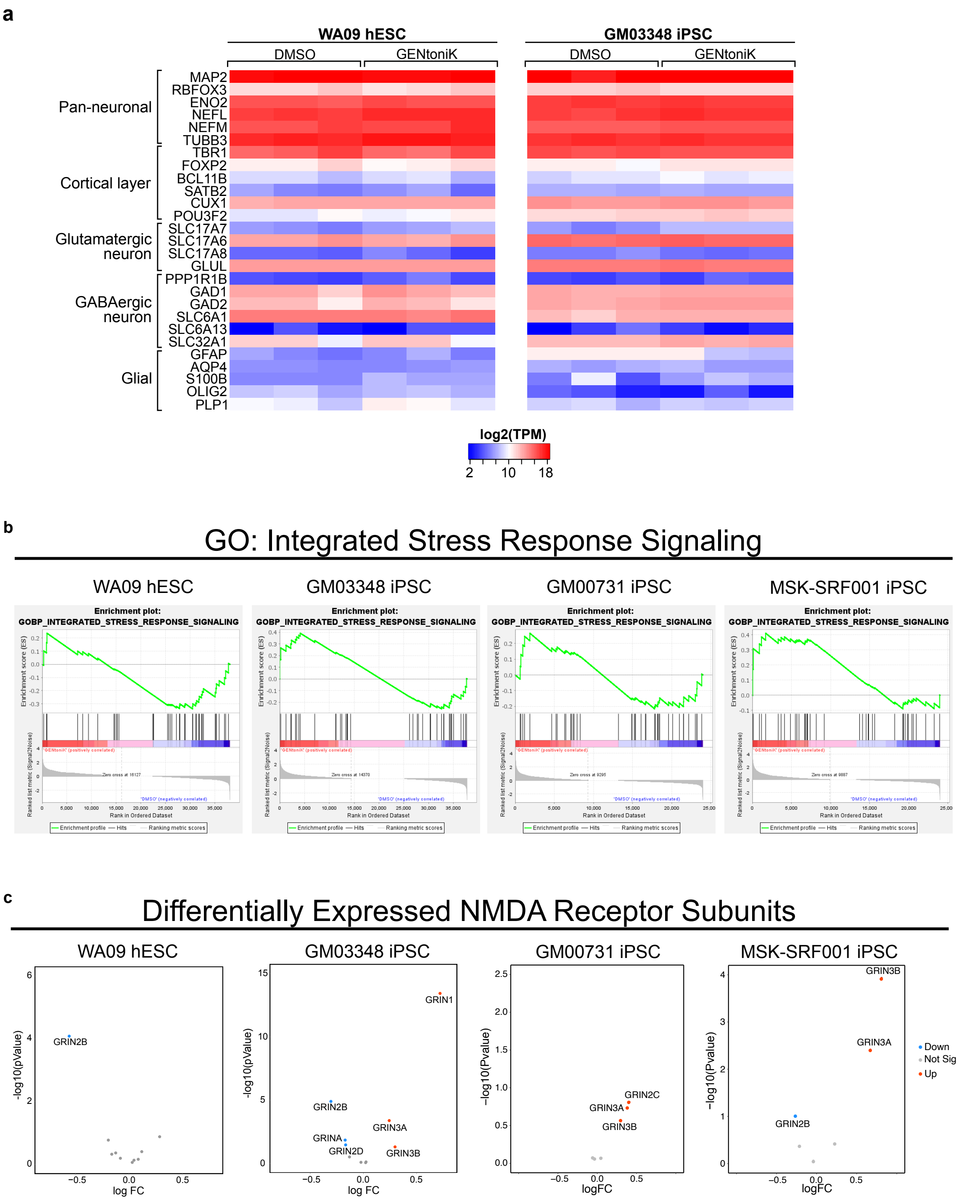

***Supplementary Figure 11 - GENtoniK induces changes in transcription without altering neuron subtype specification. a**, Bulk-RNA analysis in neurons derived from two hPSC lines reveal no drastic changes in the expression of markers of pan-neuronal identity, cortical layer, glutamatergic neuron, GABAergic neuron, or glial cells. **b**, GSEA of RNA-seq results showing GENtoniK treatment does not result in appreciable enrichment in GO:0140467 integrated stress response signaling pathways. **c**, Volcano plots of genes encoding NMDA receptor subunits in neurons from four hPSC lines. GENtoniK treatment resulted in significantly reduced expression of the fetal subunit GRIN2B in three hPSC lines, significance determined using the Wald Test.*

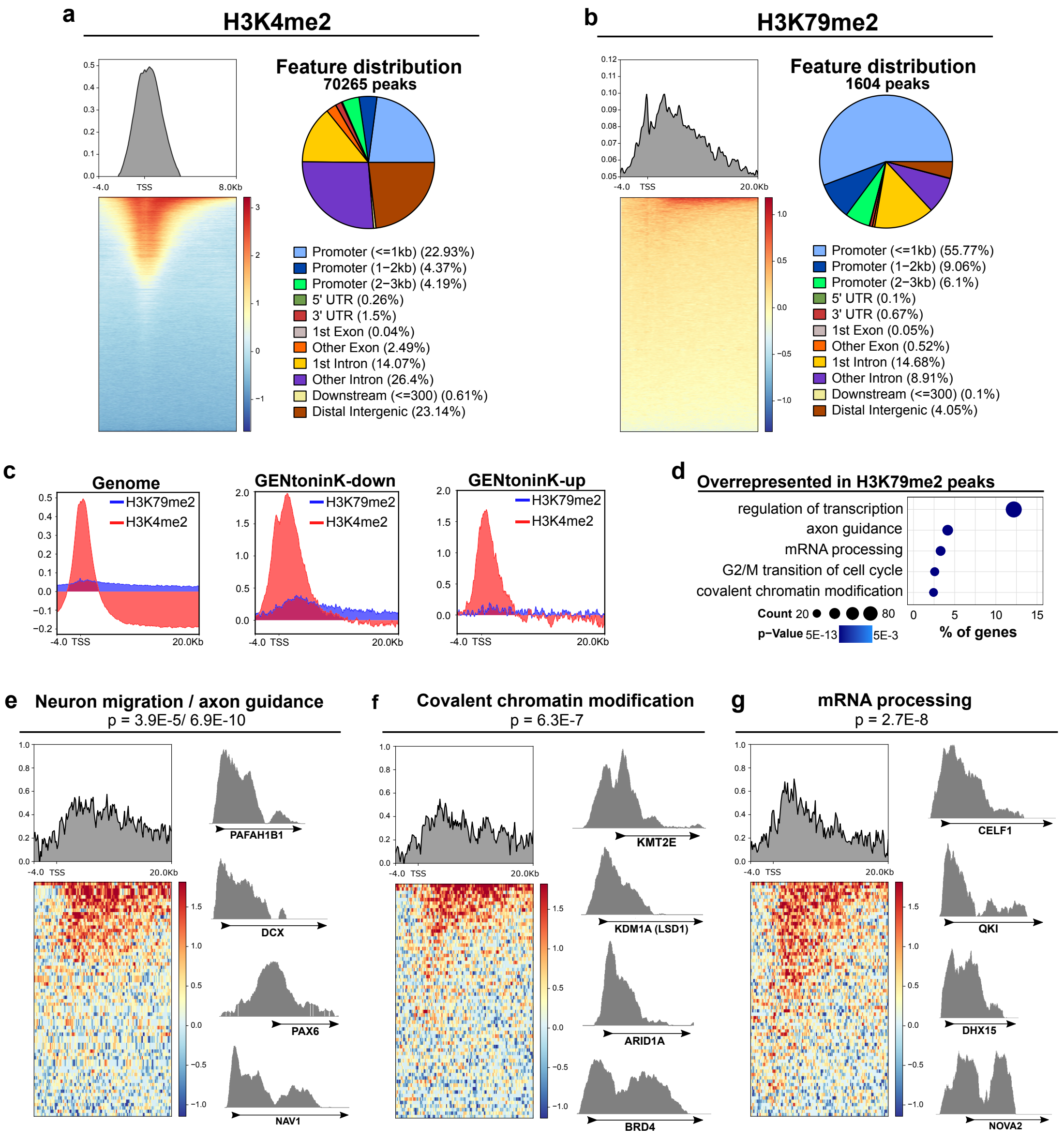

Supplementary Figure 12 - CUT&RUN analysis of LSD1 and DOT1L-targeted histone marks in untreated day 10 immature neurons. a, *Left, normalized genome enrichment profile of H3K4me2 over IGG control along 12Kb region surrounding the transcription start site (TSS). Right, genome-wide distribution of gene features among H3K4me2 peaks.* ***b****, Left, normalized genome enrichment profile of H3K79me2 over IGG control along 24Kb region surrounding the transcription start site (TSS). Right, genome-wide distribution of gene features among H3K79me2 peaks.* ***c,*** *CUT&RUN peak profiles of LSD1 and DOT1L targets H3K4 and H3K79 2-methylation in immature, untreated d7 hPSC-cortical neurons across the whole genome (left) and in genes downregulated (middle) or upregulated (right) by GENtoniK in RNA-seq.* ***d****, Gene ontology analysis showing enrichment or immature function and transcriptional regulation in genes occupied by DOT1L-target H3K79 2-methylation.* ***e-g****, Enrichment of H3K79me2 vs IGG control in gene ontology categories significantly overrepresented among H3K79me2 peaks with representative tracks for genes within each category: GO:0001764-neuron migration and GO:0007411-axon guidance (****e****), GO:0016569-covalent chromatin modification (****f****), and GO:0006397-mRNA processing (****g****). Displayed p-values are for enrichment of stated ontology categories among genes within H3K79me2 peaks, with significance determined using Fisher's Exact Test.*

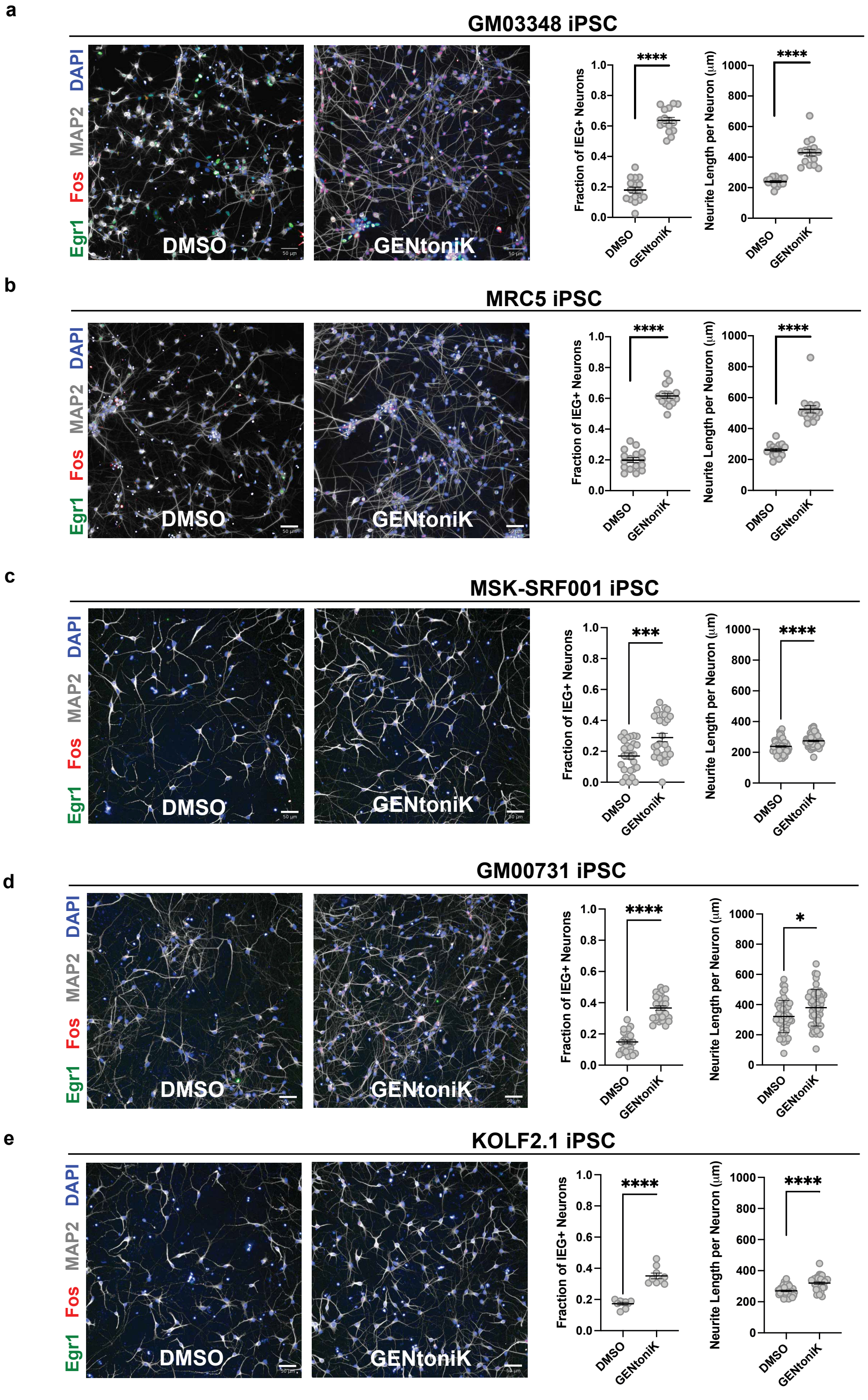

Supplementary Figure 13 - GENtoniK promotes IEG induction and neurite outgrowth of cortical neurons derived from induced pluripotent stem cells (iPSCs). a-e, *Representative high-content maturation assay images, and quantification of IEG induction by*

KCl and neurite outgrowth: ***a,*** *Neurons derived from reprogrammed normal lung fibroblast line MRC5 (n=16 microplate wells).* ***b****, Neurons derived from reprogrammed skin fibroblasts of 10-year-old male (n=16 microplate wells).* ***c,*** *Neurons derived from reprogrammed skin fibroblasts of adult female MSK-SRF001 (IEG analysis: n=27 wells both DMSO and GENtoniK; Neurite length: n=63 wells for both DMSO and GENtoniK).* ***d,*** *Neurons derived from reprogrammed skin fibroblasts of adult male GM00731 (IEG analysis: n=27 wells for both DMSO and GENtoniK; Neurite length: n=53 wells for DMSO and n=52 wells for GENtonik).* ***e,*** *Neurons derived from reprogrammed male fibroblast KOLF2.1 (IEG analysis: n=9 wells for both DMSO and GENtoniK; Neurite length: n=34 wells for DMSO and n=32 wells for GENtoniK).* ***a-e,*** *Two-tailed Welch's t-test; asterisks indicate statistical significance. Mean values are represented by a black line. Error bars represent S.E.M. Scale bars are 50 µm. Wells which exhibited peeling of monolayer basement matrix and subsequent cell loss were excluded from the analysis.*

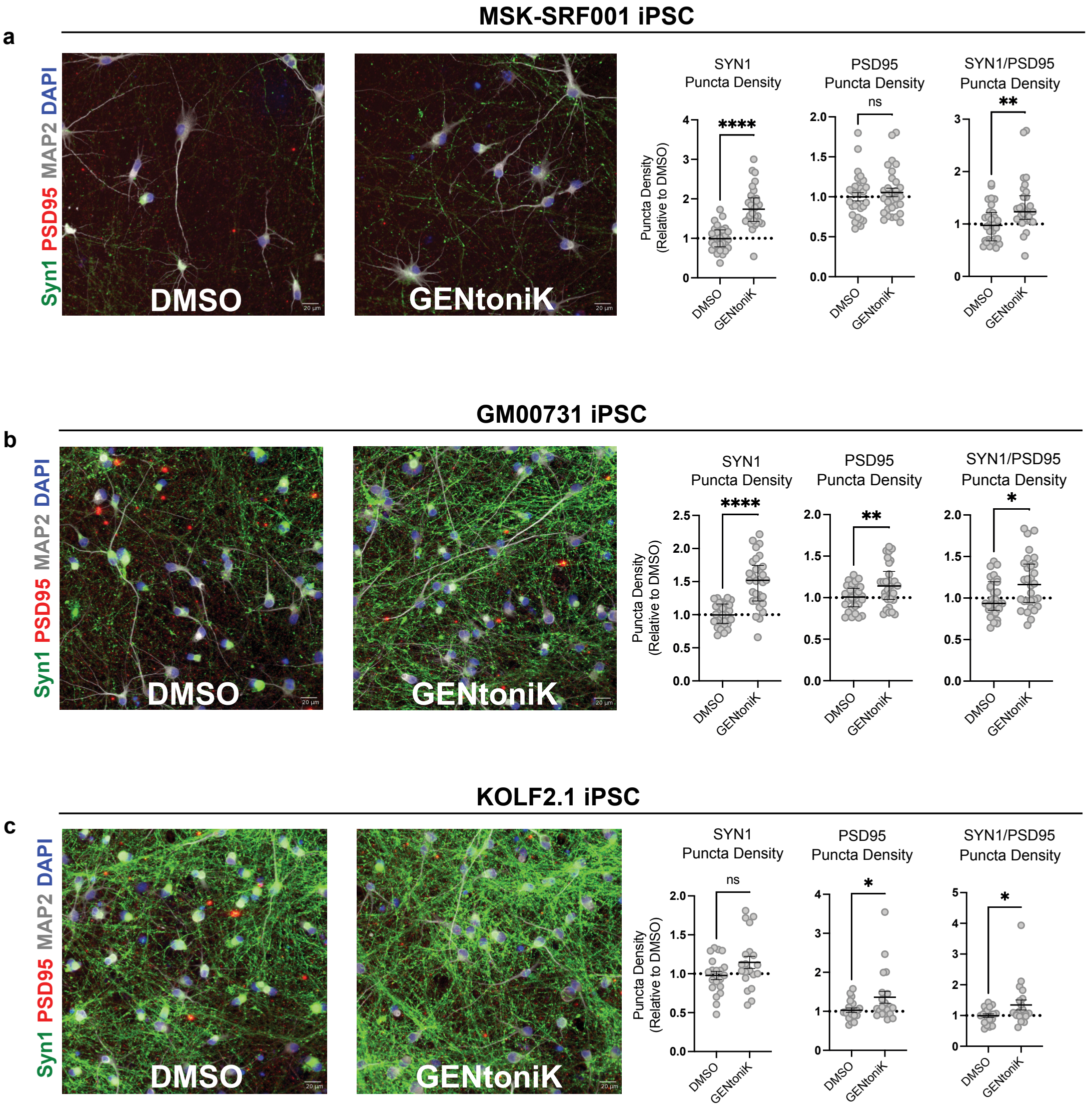

Supplementary Figure 14 - GENtoniK treatment increases assembled synapses in iPSC-cortical neurons. a-c, *Representative high-content assay images of Syn1 and PSD95 puncta density within MAP2-positive neurites, with quantification displayed relative to mean puncta density of DMSO treated conditions, represented by the black dotted line and set at 1 for each replicate.* ***a,*** *Neurons derived from reprogrammed skin fibroblasts of adult female MSK-SRF001 (n=30 wells).* ***b****, Neurons derived from reprogrammed skin fibroblasts of adult male GM00731 (n=30 wells).* ***c****, Neurons derived from reprogrammed male fibroblast KOLF2.1 (n=20 wells).* ***a-c,*** *Two-tailed Welch's t-test; asterisks indicate statistical significance. DMSO Mean values are represented by a black line. Error bars represent S.E.M. Scale bars are 20 µm.*

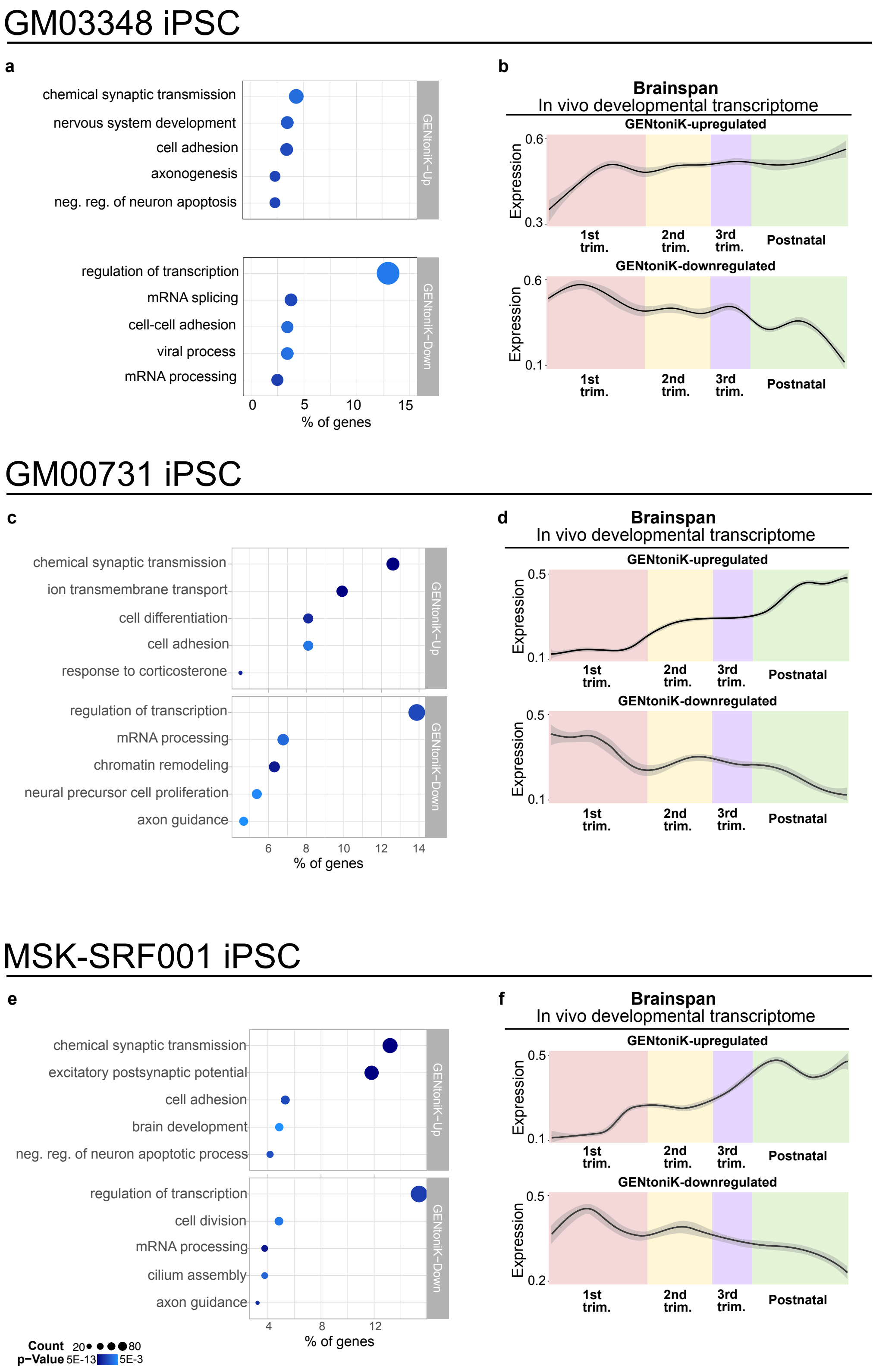

GM03348 iPSC
a
chemical synaptic transmission
nervous system development
cell adhesion
axonogenesis
neg. reg. of neuron apoptosis
GENtoniK−Up
regulation of transcription
mRNA splicing
cell-cell adhesion
viral process
mRNA processing
GENtoniK−Down
0
5
10
15
% of genes
b
Brainspan
In vivo developmental transcriptome
GENtoniK-upregulated
Expression
0.6
0.3
1st trim.
2nd trim.
3rd trim.
Postnatal
GENtoniK-downregulated
0.6
0.1
GM00731 iPSC
c
chemical synaptic transmission
ion transmembrane transport
cell differentiation
cell adhesion
response to corticosterone
GENtoniK−Up
regulation of transcription
mRNA processing
chromatin remodeling
neural precursor cell proliferation
axon guidance
GENtoniK−Down
6
8
10
12
14
% of genes
d
Brainspan
In vivo developmental transcriptome
GENtoniK-upregulated
0.5
0.1
GENtoniK-downregulated
0.5
0.1
MSK-SRF001 iPSC
e
chemical synaptic transmission
excitatory postsynaptic potential
cell adhesion
brain development
neg. reg. of neuron apoptotic process
GENtoniK−Up
regulation of transcription
cell division
mRNA processing
cilium assembly
axon guidance
GENtoniK−Down
4
8
12
% of genes
Count 20 80
p−Value 5E-13 5E-3
f
Brainspan
In vivo developmental transcriptome
GENtoniK-upregulated
0.5
0.1
GENtoniK-downregulated
0.5
0.2

Supplementary Figure 15 - GENtoniK induces maturation-associated changes in gene expression in iPSC-derived cortical neurons. a-e, *Bulk RNA-seq analysis in neurons derived from indicated iPSC lines.* ***a, c, e,*** *Gene ontology analysis showing enrichment for mature neuron function in genes upregulated by the cocktail, and enrichment for immature function and transcriptional regulation in genes downregulated by the cocktail, significance determined using Fisher's Exact Test.* ***b, d, f,*** *In the BrainSpan Atlas of the Developing Human Brain (https://www.brainspan.org), genes upregulated by GENtoniK display an average expression that increases from early development to gestation and after birth (top), genes downregulated by GENtoniK display higher average expression during early development and decrease over time (bottom). Black lines represent smoothed means curve with bands representing confidence intervals.*

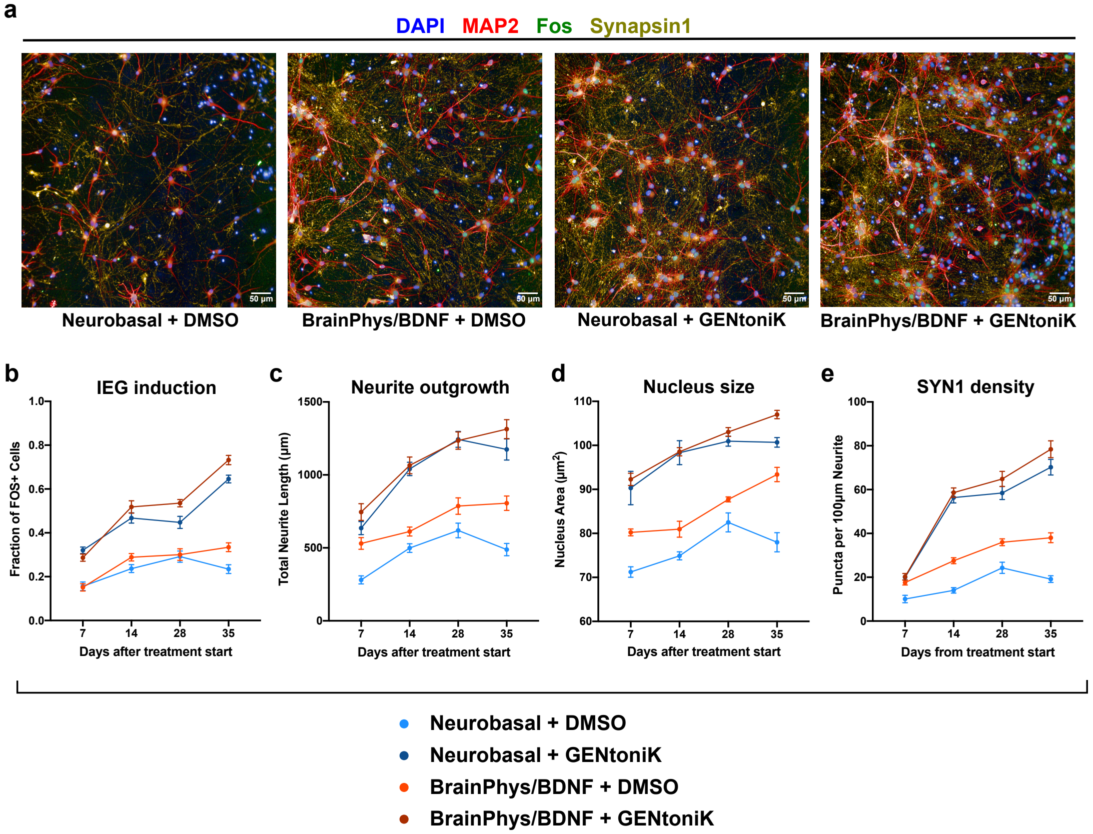

***Supplementary Figure 16 - GENtoniK improves upon and complements alternative neuron maturation strategies. a**, Immunofluorescent stain for MAP2, FOS, and SYN1 of day 35 hPSC-derived cortical neurons in plain Neurobasal medium, BrainPhys medium+BDNF, Neurobasal with GENtoniK, and BrainPhys+BDNF with GENtoniK. **b-e,** Time-course quantification of the maturity parameters: FOS induction by KCl (**b**), neurite length (**c**), nucleus size (**d**), and SYN1 puncta density (**e**) in neurons that received GENtoniK versus DMSO from day 7 from plating. Plates were collected for analysis every 7 days, beginning 7 days after the start of DMSO/GENtoniK treatment. n=12 microplate wells. Error bars represent S.E.M. Scale bars are 50 µm.*

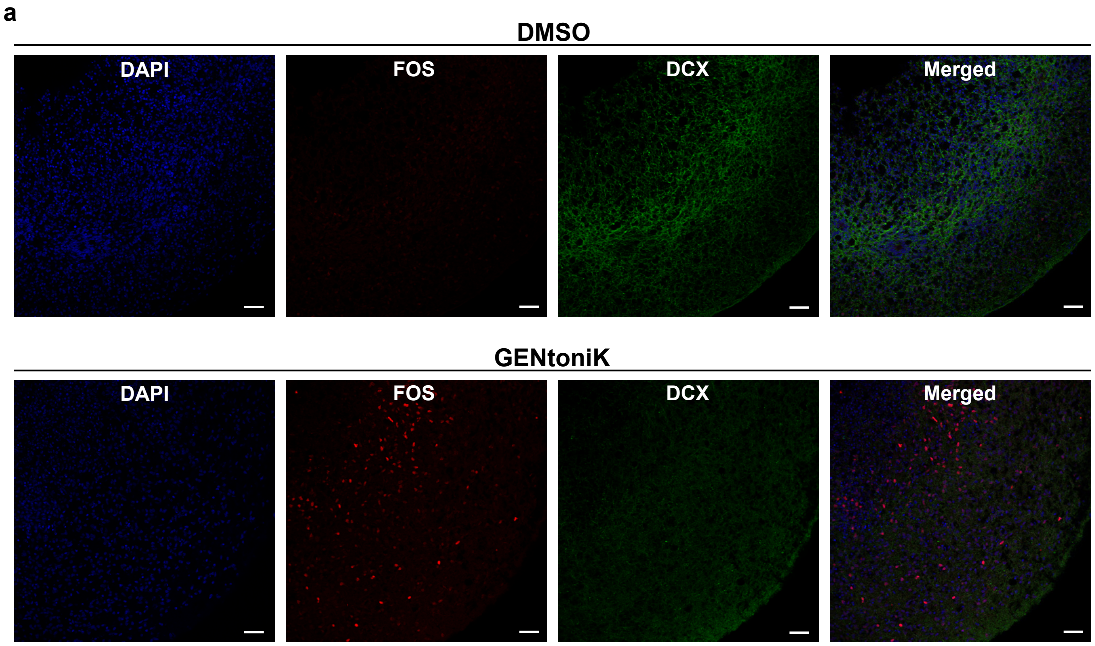

Supplementary Figure 17 - GENtoniK decreases migratory marker expression and increases neuronal activity marker expression without altering cortical layer representation in forebrain organoids. a, *Representative images of immunofluorescent staining for FOS, DCX, and MAP2 in day 60 forebrain organoids that received DMSO (top) or GENtoniK (bottom) from days 15 to 50.* ***b****, Representative images of immunofluorescent staining for deep and upper layer markers TBR1 and SATB2 in day 60 forebrain organoids that received DMSO (left) or GENtoniK (right) from days 15 to 50 (n=2 independent differentiations). Scale bars are 100 µm.*

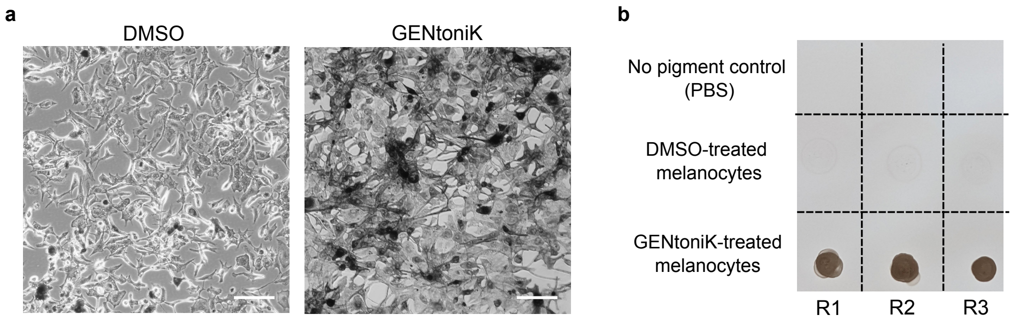

***Supplementary Figure 18 - GENtoniK treatment induces early pigmentation in hPSC-melanocytes**. **a**, Brightfield images of melanocytes (day 33 of hPSC differentiation) that received GENtoniK or DMSO from day 11. Scale Bars are 200 µm. **b**, Dot blot analysis of pigmentation in lysates of melanocytes treated with GENtoniK or DMSO (n = 3 independent differentiations). One million cells were lysed per sample and PBS was used as unpigmented control.*

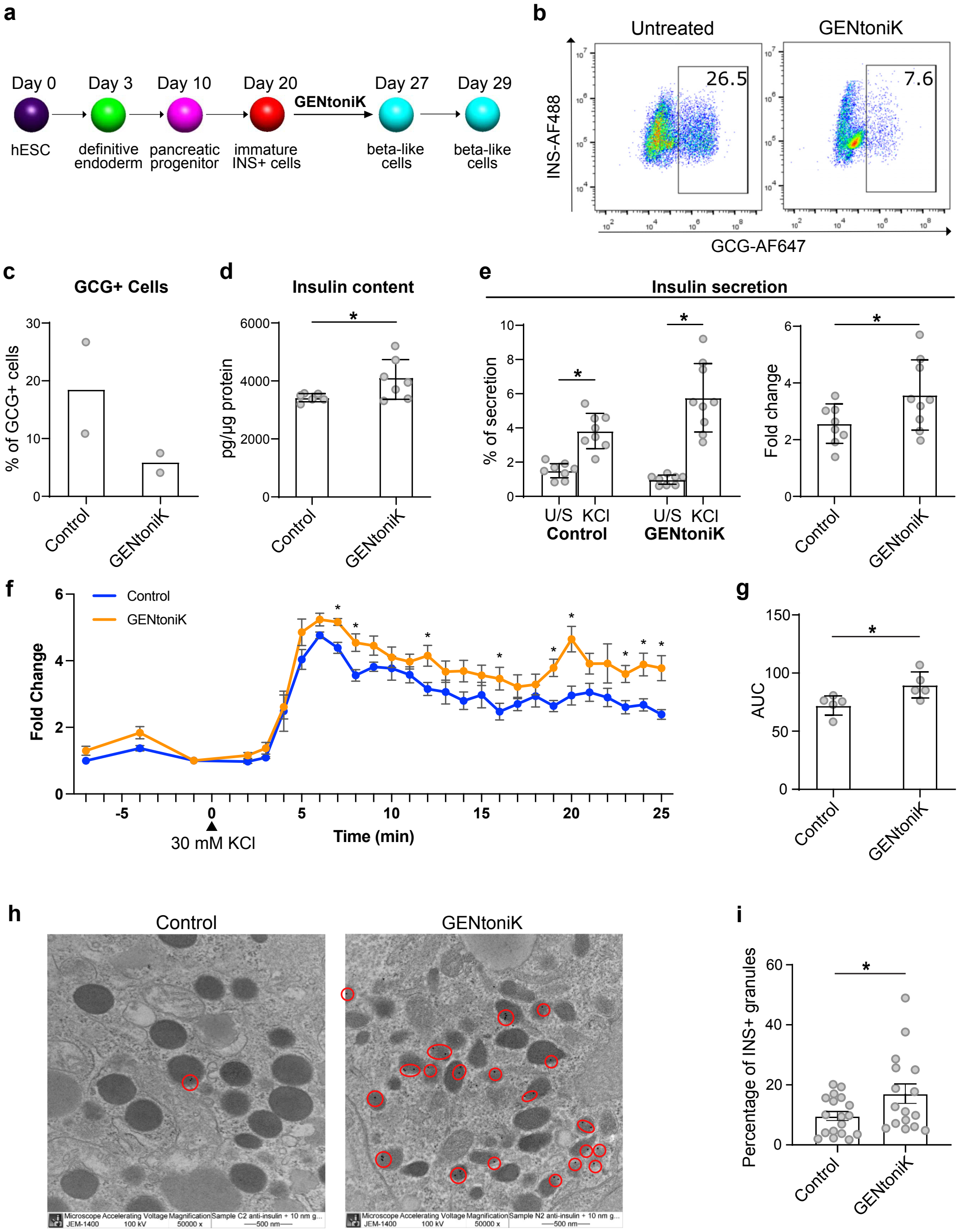

***Supplementary Figure 19 - GENtoniK promotes maturation of hESC-derived beta-like cells**. **a,** Schematic representation of the stepwise differentiation protocol. hESC-derived immature beta-like cells were treated with GENtoniK or DMSO from days 20 to 27. **b-c,** Representative flow cytometry analysis (**b**) and quantification (**c**) of the percentage of GCG+ cells in INS-GFP+ cells after 7 days treatment with GENtoniK or control followed by 2 days treatment-free (n=2 independent differentiations). **d**, Total insulin content of INS-GFP+ cells after 7 days treatment with GENtoniK or control followed by 2 days treatment-free culture (n=6 biological replicates for control and n=7 for GENtoniK). **e**, Static KCl-stimulated human insulin secretion and fold change in beta-like cells after 7 days treatment with GENtoniK or control followed by 2 days treatment-free culture. The assay was performed in the presence of 2 mM D-glucose (n=8 biological replicates for unstimulated control, KCL-stimulated control, and unstimulated GENtonik conditions, and n=9 for KCL-stimulated GENtoniK). **f-g,** Dynamic KCl stimulated human insulin secretion (**f**) and area*

*under curve (AUC, **g**) in hESC-derived cells after 7 days treatment with GENtoniK or control followed by 2 days treatment-free culture. The assay was performed in the presence of 2 mM D-glucose. Fold change was calculated by dividing the amount of secreted insulin at each time point by the average amount of secreted insulin at 2 mM D-glucose (n=5 biological replicates per group). **h**, Representative electron micrographs showing immunogold labelling of insulin in beta-like cells. Circles indicate insulin^{+} granules (10 nm gold particles). Magnification=50,000x. **i**, Percentage of insulin^{+} granules in control and GENtoniK-treated beta-like cells (n=18 for DMSO and n=16 for GENtoniK). **c** and **e**, Two-tailed Student's t-test; asterisks indicate statistical significance. Error bars represent S.E.M.*

hPSC Neuron Gating Strategy

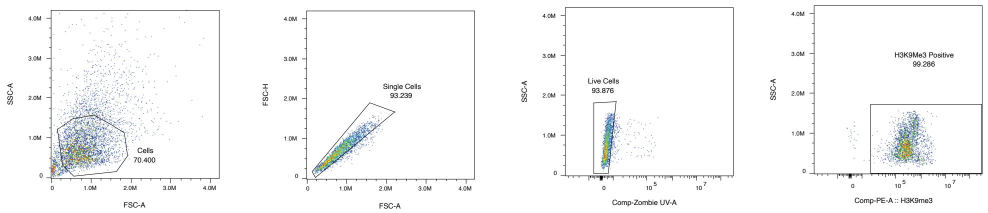

Supplementary Figure 20 - Gating Scheme for flow cytometry analysis of hPSC neurons. *FSC-A/SSC-A were used to remove the debris and cell fragments. FSC-A/FSC-H was used to remove doublets. Zombie-UV/SSC-A were used to remove dead cells.*
