# Supplementary material for: Combined small-molecule treatment accelerates maturation of human pluripotent stem cell-derived neurons
Source: Nat Biotechnol. 2024 Jan 2;42(10):1515–25. doi: 10.1038/s41587-023-02031-z (PMC11348887; doi:10.1038/s41587-023-02031-z)
Supplement: Supplementary file 2 — Reporting Summary [file 41587_2023_2031_MOESM2_ESM.pdf]

Corresponding author(s): Lorenz Studer
Last updated by author(s): Aug 11, 2023

Reporting Summary

Nature Research wishes to improve the reproducibility of the work that we publish. This form provides structure for consistency and transparency in reporting. For further information on Nature Research policies, see our Editorial Policies and the Editorial Policy Checklist.

Statistics

For all statistical analyses, confirm that the following items are present in the figure legend, table legend, main text, or Methods section.

n/a	Confirmed	
☐	☒	The exact sample size (*n*) for each experimental group/condition, given as a discrete number and unit of measurement
☐	☒	A statement on whether measurements were taken from distinct samples or whether the same sample was measured repeatedly
☐	☒	The statistical test(s) used AND whether they are one- or two-sided<br>*Only common tests should be described solely by name; describe more complex techniques in the Methods section.*
☒	☐	A description of all covariates tested
☒	☐	A description of any assumptions or corrections, such as tests of normality and adjustment for multiple comparisons
☒	☐	A full description of the statistical parameters including central tendency (e.g. means) or other basic estimates (e.g. regression coefficient) AND variation (e.g. standard deviation) or associated estimates of uncertainty (e.g. confidence intervals)
☐	☒	For null hypothesis testing, the test statistic (e.g. F, t, r) with confidence intervals, effect sizes, degrees of freedom and P value noted<br>*Give P values as exact values whenever suitable.*
☒	☐	For Bayesian analysis, information on the choice of priors and Markov chain Monte Carlo settings
☒	☐	For hierarchical and complex designs, identification of the appropriate level for tests and full reporting of outcomes
☒	☐	Estimates of effect sizes (e.g. Cohen's *d*, Pearson's *r*), indicating how they were calculated

Our web collection on statistics for biologists contains articles on many of the points above.

Software and code

Policy information about availability of computer code

Data collection	Zeiss ZEN was used for image acquisition. Electrophysiology recordings were performed using pClamp 10 (single cell) and BrainWave 4 (MEA).
Data analysis	High-content image analysis was performed using PerkinElmer Columbus software 2.9.0.1546. Downstream normalization and analysis were performed using the KNIME 3.6 platform with HCS tools module. Statistical tests were performed using GraphPad Prism 9.1. RNA-seq and CUT&RUN analyses were performed in the Galaxy platform. FASTQ files were mapped using Salmon (RNA-seq) or Bowtie 2 (CUT&RUNW, GS). DEseq2 was used for differential expression analysis. CUT&RUN peak calling and visualization were performed using MACSC, hlPSeeke,ra , nd deepTools2. Copy number aberration analysis was conducted using the CNAclinic package in R. R software analysis was done in version 3.5 or later. Live cell imaging analysis was done using the Inccucyte version 2020A.

For manuscripts utilizing custom algorithms or software that are central to the research but not yet described in published literature, software must be made available to editors and reviewers. We strongly encourage code deposition in a community repository (e.g. GitHub). See the Nature Research guidelines for submitting code & software for further information.

Data

Policy information about availability of data

All manuscripts must include a data availability statement. This statement should provide the following information, where applicable:

- Accession codes, unique identifiers, or web links for publicly available datasets
- A list of figures that have associated raw data
- A description of any restrictions on data availability

Data generated during this study are deposited at NCBI GEO under accession numbers GSE172544 (RNA-seq) and GSE172543 (CUT&RUN). GRCh37 hg19 is publicly available at https://www.ncbi.nlm.nih.gov/datasets/genome/GCF_000001405.13/ and GRCh37 p13 is available at https://www.ncbi.nlm.nih.gov/datasets/genome/GCF_000001405.39/.

Field-specific reporting

Please select the one below that is the best fit for your research. If you are not sure, read the appropriate sections before making your selection.

☒ Life sciences ☐ Behavioural & social sciences ☐ Ecological, evolutionary & environmental sciences

For a reference copy of the document with all sections, see nature.com/documents/nr-reporting-summary-flat.pdf

Life sciences study design

All studies must disclose on these points even when the disclosure is negative.

Sample size	A minimum of 3 biological replicates were analyzed for the study, with the sole exception of HD-MEA (n=2) where the number of electrodes per probe (4096) compensates for probe to probe variability of conventional MEAs. For technical feasibility, initial screen was performed in one line, while validation was performed in at least 3 lines with 3 independent diff. each.
Data exclusions	No samples were excluded. However, wells with technical failure (extensive monolayer pealing or non-neural contaminating cell populations) were excluded from analysis.
Replication	Excluding technical issues, findings were reproduced in at least 3 biological replicates. Where specified, replication was extended to additional pluripotent lines from different sexes and derivations. Cell lines which failed to produce pure neuronal populations, or experiments where cells detached from plate surface were not used as replicates (see above).
Randomization	Screening compounds were organized according to library layout. During validation, samples were randomly assigned treatment conditions.
Blinding	Investigators were not blinded but screening data collection and hit selection were conducted in an automated manner.

Reporting for specific materials, systems and methods

We require information from authors about some types of materials, experimental systems and methods used in many studies. Here, indicate whether each material, system or method listed is relevant to your study. If you are not sure if a list item applies to your research, read the appropriate section before selecting a response.

Materials & experimental systems

n/a	Involved in the study	
☐	☒	Antibodies
☐	☒	Eukaryotic cell lines
☒	☐	Palaeontology and archaeology
☒	☐	Animals and other organisms
☒	☐	Human research participants
☒	☐	Clinical data
☒	☐	Dual use research of concern

Methods

n/a	Involved in the study	
☐	☒	ChIP-seq
☐	☒	Flow cytometry
☒	☐	MRI-based neuroimaging

Antibodies

Antibodies used

Antigen Supplier Catalog # Host species
c-Fos Abcam ab208942 Mouse
c-Fos Cell Signaling Technology 2250 Rabbit
EGR1 Cell Signaling Technology 4153 Rabbit
MAP2 Abcam ab5392 Chicken
Synapsin 1 Cell Signaling Technology 5297 Rabbit
PSD95 Abcam ab2723 Mouse
TUJ1 (β3-tubulin) Abcam ab107216 Chicken
ISL1/2 DSHB 39.4D5 Mouse
DCX Cell Signaling Technology 4604 Rabbit
TBR1 Abcam ab183032 Rabbit
FOXG1 (BF-1) Takara Bio M227 Mouse
H3K4me2 Upstate 07-030 Rabbit
H3K79me2 Active Motif 39143 Rabbit
Mouse IgG Abcam ab46540 Rabbit
CD117 (c-Kit) Invitrogen 17-1179-42 Mouse
Insulin Dako A0564 Guinea pig
Glucagon Abcam ab189279 Rabbit
Tau Dako A0024 Rabbit
4-repeat Tau Cell Signaling Technology 30328 Rabbit
GAPDH Santa Cruz sc-47724 Mouse
H3K9me3 Abcam ab8898 Rabbit

H3K9ac Abcam ab4441 Rabbit
H3K9me3 (PE Conjugate) Cell Signaling Technology 55286 Rabbit
10 nm Gold Goat Anti Guinca Pig IgG - Aurion SKU: 810.144

Validation

Abcam ab208942: validated for ICC and WB in human tissue by manufacturer. Validated in FOS cell knockout line by manufacturer. A widely used antibody for immunofluorescence, it appears in 75 citations on manufacturers website and 127 citations of Citeab website.
CST 2250: validated for ICC and WB in mouse cortical tissue and human tissue by manufacturer. Widely used antibody for detection of c-Fos, appearing in 773 citations on manufacturer website and 763 on Citeab website.
CST 4153: validated for ICC and WB in human cells and mouse brain tissue by manufacturer. Widely used antibody for detection of EGR1, appearing in 160 citations of maunfacturer website and in 154 citations on Citeab Website.
Abcam ab5392: generated with human antigen. Validated for ICC and WB in mouse and rat brain tissue by manufacturer. Widely used in immunofluorescence, cited in 681 references on manufacturer website and 904 citations on Citeab website.
CST 5297: validated for ICC and WB in human cerebellum tissue by manufacturer. Widely used for detection of Synanpsin-1, cited in 159 references on the manufacturer website and 151 references on Citeab.
Abcam ab2723: validated for ICC and WB in mouse and rat brain tissue by manufacturer. Human reactivity confirmed by secondary-only control and colocalization with other compartment-specific proteins. Widely used for detection of PSD-95 including by immunofluorescence. Cited in 216 references on Citeab and 169 references on manufacturer website.
Abcam ab107216: Validated in rat brain tissue and knockout validated in human cell line by manufacturer for WB and ICC. Appears in 41 references according to archived product data sheet from manufacturer website.
DSHB 39.4D5: ICC reactivity tested by depositors (see Sanders et al., 2003, and Georgia et al., 2006). Widely cited for IF detection of ISL1/2. Appears in 628 citations according to Citeab website.
CST 4604: validated for ICC and WB in rodent tissue by manufacturer. Human reactivity confirmed by secondary-only control. Widely cited for detecting Doublecortin, appears in 264 citations according to the manufacturer and 261 citations on CiteAb website.
Abcam ab183032: validated for ICC and WB in rodent brain, as well as human cerebral organoid tissue and in human brain lysate, by manufacturer. Widely used for detection of TBR-1 by immunofluorescence. Appears in 14 references on the manufacturer website and 30 citations according to Citeab website.
Takara Bio M227: generated with human antigen. Validated for ICC in rodent tissue by manufacturer. Appears in at least 4 references according to Takara Website.
Upstate 07-03: Specificity demonstrated by ELISA using methylated peptides and validated for Ch IP-seq in human cells by manufacturer
Active Motif 39143: validated for ChIP-seq in human cells by manufacturer. Appears in 15 citations according to CiteAb website.
Abcam ab46540: used as negative control (see Skene & Henikoff, 2017), is widely used in 343 citations according to CiteAb website.
Invitrogen 17-1179-42: validated for flow cytometry in human pluripotent stem cell-derived cells by manufacturer. Used in 13 citations according to CiteAb website.
Dako A0564: validated in Beta cells in islets of Langerhans in human pancreas by manufacturer. Widely used in detection of Insulin, cited in 642 references as per CiteAb website.
Abcam ab189279: validated in cells of islets of Langerhans in human pancreas, and by flow cytometry in human cells lines, by manufacturer. Has appeared in one reference according to manufacturer and CiteAb website.
Cell Signaling Technology 55286: validated for flow cytometry in human cell lines by manufacturer. Non conjugated clone (CST 13969) of the antibody is widely used and appears in 127 citations on manufacturer website and 126 on CiteAb website.

Eukaryotic cell lines

Policy information about cell lines

Cell line source(s)

H9 (WA-09): WiCell Stemcell Bank. MEL-1: Stem Cell Sciences Ltd. GM03348 iPSC, GM00731 iPSC, and MRC-5 iPSC: Coriell. MSK-SRF00l iPSC: Memorial Sloan Kettering Institute. KOLF2.1: Jackson Laboratory. Mel-1 is from Stem Cell Sciences LTD.

Authentication

hESCs were authenticated by STR. iPSCs have not been authenticated.

Mycoplasma contamination

All cell lines are regularly tested for mycoplasma contamination and were negative

Commonly misidentified lines
(See ICLAC register)

No commonly misidentified lines were used in this study.

ChIP-seq

Data deposition

☒ Confirm that both raw and final processed data have been deposited in a public database such as GEO.

☒ Confirm that you have deposited or provided access to graph files (e.g. BED files) for the called peaks.

Data access links<br>*May remain private before publication.*	https://www.ncbi.nlm.nih.gov/geo/query/acc.cgi?acc=GSE172543
Files in database submission	H3K79me2Peaks.csv, H3K4me2Peaks.csv, H3K79me2_01_R1.fastq.gz, H3K79me2_02_R1.fastq.gz, H3K79me2_03_R1.fastq.gz, H3K79me2_01_R2.fastq.gz, H3K79me2_02_R2.fastq.gz, H3K79me2_03_R2.fastq.gz, H3K4me2_01_R1.fastq.gz, H3K4me2_02_R1.fastq.gz, H3K4me2_03_R1.fastq.gz, H3K4me2_01_R2.fastq.gz, H3K4me2_02_R2.fastq.gz, H3K4me2_03_R2.fastq.gz, ControlIGG_01_R1.fastq.gz, ControlIGG_02_R1.fastq.gz, ControlIGG_03_R1.fastq.gz, ControlIGG_01_R2.fastq.gz, ControlIGG_02_R2.fastq.gz, ControlIGG_03_R2.fastq.gz
Genome browser session<br>(e.g. UCSC)	http://genome.ucsc.edu/s/ejhergenreder/H3K4me2H3K79me2

Methodology

Replicates	CUT&RUN experiment. 3 biological replicates from independent differentiations per antibody.
Sequencing depth	Paired-end. 10-17M reads per sample, >70% uniquely aligned. Note lower depth requirement for CUT&RUN vs ChIP-seq
Antibodies	H3K79me2 (Active Motif 39143), H3K4me2 (Upstate 07-030), Mouse IGG background control (abcam ab46540)
Peak calling parameters	Peaks were called using MACS: lower mfold (5), upper mfold (50), band width (180). Broad peaks were called for H3K79me2 with broad region cutoff (0.1)
Data quality	Peaks with a q-value cutoff of 0.05 were used for downstream analysis
Software	Bowtie2 was used for alignment. Peaks were called using MACS. Analysis and visualization were performed using ChIPSeeker, and deepTools2.

Flow Cytometry

Plots

Confirm that:

☒ The axis labels state the marker and fluorochrome used (e.g. CD4-FITC).

☒ The axis scales are clearly visible. Include numbers along axes only for bottom left plot of group (a 'group' is an analysis of identical markers).

☒ All plots are contour plots with outliers or pseudocolor plots.

☒ A numerical value for number of cells or percentage (with statistics) is provided.

Methodology

Sample preparation	hESC-derived beta-like cells were dissociated using Accutase, fixed and permeabilized using BD Biosciences Fixation/ Permeabilization Solution Kit.<br><br>hPSC-neurons were dissociated using Accutase (Innovative Cell Technologies) supplemented with Neuron Isolation Enzyme for Pierce™ (Thermo), stained with Zombie UV™ Fixable Viability Kit (Biolegend), fixation in 4% Paraformaldehyde, permeabilized in 0.5% triton-x, and blocked in 5% BSA.
Instrument	Accuri C6 (Beta-like cells), Cytek Aurora (neurons)
Software	FlowJo v10
Cell population abundance	200,000 cells were collected (Beta-like Cells), 100,000 cells were collected (Neurons)
Gating strategy	(Beta Cells) FSC-A/SSC-A were used to remove the debris and cell fragments. FL1 was applied to gate the INS-Alexa-488+ cells. Finally, FL-4 was applied to gate the GCG-Alexa-647+ cells among INS-Alexa-488+ cells.<br><br>(Neurons) FSC-H/SSC-H were used to remove the debris and cell fragments. FSC-A/FSC-H was used to remove doublets. Zombie-UV/SSC-A were used to remove dead cells.

☒ Tick this box to confirm that a figure exemplifying the gating strategy is provided in the Supplementary Information.
